# Design, synthesis, and biological investigation of new thiazole-based derivatives as multi-targeted inhibitors endowed with antiproliferative, antioxidant, and antibacterial properties

**DOI:** 10.3389/fchem.2025.1595997

**Published:** 2025-05-06

**Authors:** Hesham A. M. Gomaa, Asmaa M. Atta, Mohamed E. Shaker, Sami I. Alzarea, Tariq G. Alsahli, Eid Alatwi, Arafa Musa, Fatma A. M. Mohamed, Hayat Ali Alzahrani, Aliaa M. Mohassab, Alshaimaa Abdelmoez, Stefan Bräse, Bahaa G. M. Youssif

**Affiliations:** ^1^ Department of Pharmacology, College of Pharmacy, Jouf University, Sakaka, Aljouf, Saudi Arabia; ^2^ Pharmaceutical Chemistry Department, Faculty of Pharmacy, Badr University in Cairo (BUC), Badr City, Cairo, Egypt; ^3^ Department of Pharmacognosy, College of Pharmacy, Jouf University, Sakaka, Aljouf, Saudi Arabia; ^4^ Department of Clinical Laboratory Sciences, College of Applied Medical Sciences at Al-Qurayyat, Jouf University, Al-Qurayyat, Saudi Arabia; ^5^ Applied Medical Science College, Medical Laboratory Technology Department, Northern Border University, Arar, Saudi Arabia; ^6^ Department of Medicinal Chemistry, Faculty of Pharmacy, Minia University, Minia, Egypt; ^7^ Department of Pharmaceutical Organic Chemistry, Faculty of Pharmacy, Assiut University, Assiut, Egypt; ^8^ Institute of Biological and Chemical Systems, IBCS-FMS, Karlsruhe Institute of Technology, Karlsruhe, Germany

**Keywords:** anticancer, antioxidant, antibacterial, EGFR, VEGFR-2

## Abstract

**Introduction:**

A novel series of thiazole-based derivatives **11a-f** and **12a-f** was developed, synthesized, and tested for antiproliferative activity as dual EGFR/VEGFR-2 inhibitors, antioxidants, and antibacterial agents.

**Methods:**

The structures of the new compounds **11a-f** and **12a-f** were validated using NMR spectroscopy and elemental microanalysis. The antiproliferative activity of **11a-f** and **12a-f** was tested against a panel of four cancer cell lines using MTT assay.

**Results and Discussion:**

Compounds **11d** and **11f** had the highest antiproliferative activity, with GI_50_ values of 30 and 27 nM, respectively, making them more potent than erlotinib (GI_50_ = 33 nM). Inhibitory studies for EGFR and VEGFR-2 demonstrated that compounds **11d** and **11f** were the most potent derivatives with dual inhibitory activity. Furthermore, compounds **11d** and **11f** exhibited significant antioxidant activity at 10 μM, with radical scavenging activity of 71% and 73%, respectively, compared to the reference Trolox (78%). Moreover, compounds **11a-f** and **12a-f** exhibit significant inhibitory activity against *E. coli* DNA gyrase, with compounds **11b**, **11e**, and **12b** displaying the highest inhibitory efficacy, yielding IC_50_ values of 182, 190, and 197 nM, respectively, in comparison to the reference novobiocin (IC_50_ = 170 nM). Compounds **11b** and **11e** have significant antibacterial efficacy against both Gram-positive and Gram-negative bacterial strains, as demonstrated by a twofold serial dilution experiment. They exhibit similar efficacy against *S. aureus, E. coli*, and *P. aeruginosa*, demonstrating more potency than ciprofloxacin, however displaying reduced effectiveness against *B. subtilis* compared to ciprofloxacin.

## 1 Introduction

**FIGURE 1:**
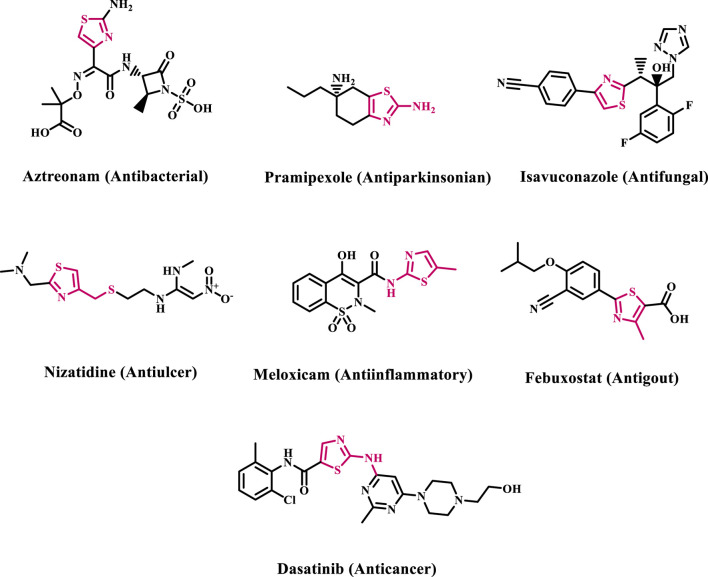
Structures of some clinically approved thiazole-based drugs.

Nitrogen-containing heterocyclic molecules are crucial in drug development, with around 75% of FDA-approved small-molecule medicines comprising one or more nitrogen-based heterocycles (Al-Wahaibi et al., 2024a; Mohamed et al., 2024). Thiazole, also known as 1,3-thiazole, is an azole heterocyclic moiety that includes one sulfur and one nitrogen atom at positions one and 3, respectively. Their varied biological activity is evidenced by numerous clinically authorized thiazole-containing drugs exhibiting various pharmacological effects. Most of these compounds are 2,4-disubstituted thiazole derivatives, with just a limited number being 2,5-disubstituted or 2,4,5-trisubstituted thiazoles (Arshad et al., 2022; Alateeq et al., 2024; Abdelazeem et al., 2017; Abdel-Aziz et al., 2021; Abdel‐Aziz et al., 2022). Several medicines having antibacterial, antiparkinsonian, antithrombotic, antifungal, antiulcer, anti-inflammatory, anticancer, antiparasitic, and antigout action contain one thiazole moiety in their structure (Figure 1) (Jadhav et al., 2021; Chugh et al., 2022).

Enzyme inhibition has recently been recognized as a vital and important target method for tumor treatment (Liu et al., 2024; Wang et al., 2021). Thiazole-based derivatives in cancer therapy have been shown to efficiently inhibit many enzymes and enzymatic pathways, including tyrosine kinase inhibitors, B-RAF enzyme inhibitors, and microtubule function suppression (Rana et al., 2023; Sharma et al., 2020). Several risk factors contribute to molecular variations or mutations in critical proteins, such as tyrosine kinases, initiating carcinogenesis. These tyrosine kinases play crucial roles in proper cell proliferation, differentiation, metabolism, migration, and cell-cycle regulation by phosphorylating tyrosine residues in proteins (Theivendren et al., 2021; Pang et al., 2022). Conversely, receptor tyrosine kinases (RTKs) are high-affinity cell surface receptors that facilitate transmembrane signalling and play a transformative role in a variety of malignancies (Batool et al., 2023; Qi et al., 2024). The FDA (Food and Drug Administration) has approved numerous medications to treat cancer caused by activated RTKs (Zhong et al., 2021; Kumar et al., 2024).

Epidermal Growth Factor Receptor (EGFR) is a type of membrane RTK that is overexpressed in many cancers. Cancer progression is intimately tied to EGFR TK signal transmission, therefore blocking receptor activation can effectively stop tumor growth (Al-Wahaibi et al., 2023a; Al-Wahaibi et al., 2024b). VEGFR-2 (Vascular Endothelial Growth Factor Receptor) is a RTK that can promote angiogenesis (Marzouk et al., 2020). VEGFR-2, a member of the VEGFR family, regulates the proliferation of blood vessels in tumors and is required to form solid tumors. Blocking VEGFR-2 has been proposed as a potential method to reduce angiogenesis (Mahmoud et al., 2023; Mahmoud et al., 2024).

EGFR and VEGFR-2 have been identified as viable therapeutic targets in cancer treatment. They are essential in signalling networks that regulate tumor cell angiogenesis, motility, differentiation, and proliferation (Al-Wahaibi et al., 2024a; Xie et al., 2018). EGFR and VEGFR-2 often share similar signalling pathways in a complicated network of interconnections. Inhibiting EGFR can diminish VEGF synthesis and obstruct angiogenesis. Simultaneously increasing VEGFR-2 expression may ultimately result in resistance to EGFR treatments. Thus, the concurrent inhibition of both EGFR and VEGFR-2 has arisen as an effective cancer therapeutic strategy, producing a synergistic impact (Liu Z.-L. et al., 2023; Liu X.-J. et al., 2023).

Bacterial infections, caused by Gram-positive or Gram-negative bacteria, are the predominant form of infections contracted in hospitals or by the general populace (Al-Wahaibi et al., 2024c; Al-Wahaibi et al., 2024d). Furthermore, bacteria have acquired resistance to almost all presently utilized antibiotics due to their prolonged, extensive, and improper application, intensifying the issue (Al-Wahaibi et al., 2024e). Each year, drug-resistant infections kill approximately 0.7 million people worldwide, a figure that might rise to 10 million by 2050 if current trends continue (Agrawal and Patel, 2024). Consequently, it is critical to speed up the development of new antibacterials that are highly efficient against both susceptible and resistant pathogens.

DNA gyrase, a topoisomerase II enzyme, modifies the topology of DNA. It has two parts, called GyrA and GyrB, that are important for separating two DNA strands and starting a process called negative supercoiling during DNA replication (Abdel-Aziz et al., 2023; Al-Wahaibi et al., 2021). Antibacterial medications that particularly target DNA gyrase work through two mechanisms: gyrase poisoning, as reported with Ciprofloxacin, and inhibiting the ATP binding site, as demonstrated in Novobiocin (Tiz et al., 2019). Because of its profound relevance, DNA gyrase has emerged as a fascinating target for developing antibacterial medications.

The prevalence of cancer-related mortality as well as new cases associated with treatment or chronic infections highlight the link between infection and cancer. Infectious agents including bacteria and viruses cause approximately 2 million new cancer cases (Seong et al., 2025; Portela et al., 2025). Individuals with persistent infections are more likely to develop cancer because their immune systems are unable to battle both the pathogen and the development of cancer cells (Woo et al., 2025). This weakness may also result from overly aggressive cancer treatments such as chemotherapy, radiotherapy, and surgical resection, which make patients susceptible to pathogenic infections. Furthermore, chronic infection causes inflammation, which contributes to the development of cancer (Zolfi et al., 2025).

**FIGURE 2:**
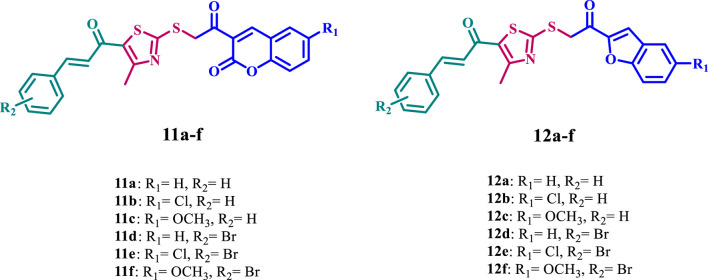
Structures of new compounds **11a-f** and **12a-f**.

Considering these precedents, this report presents an efficient synthesis of a new series of thiazole-based derivatives hybridized with coumarin or benzofuran (**11a-f** and **12a-f**, Figure 2), in line with our research objectives of developing innovative methodologies for synthesizing heterocyclic systems with promising pharmacological properties (Al-Wahaibi et al., 2023b; Al-Wahaibi et al., 2023c; Aly et al., 2024; Aly et al., 2023; Elbastawesy et al., 2015; El-Sheref et al., 2023; Frejat F. O. A. et al., 2022). Furthermore, we investigated the biological activities of the newly synthesized compounds, which have potential anticancer, antibacterial, and antioxidant effects.

The newly synthesized compounds were examined for their impact on cell viability in a normal cell line to evaluate their safety profile. The antiproliferative efficacy of novel compounds was evaluated against a panel of four different cancer cell lines. The most effective compounds were assessed for their dual inhibition of EGFR and VEGFR-2, in addition to their antioxidant properties. All newly synthesized compounds were evaluated as DNA gyrase inhibitors, and the most effective derivatives were examined for their antibacterial activity against Gram-positive bacteria (*S. aureus* and *B. subtilis*) and Gram-negative bacteria (*E. coli* and *P. aeruginosa*).

## 2 Results and discussion

### 2.1 Chemistry

**SCHEME 1:**
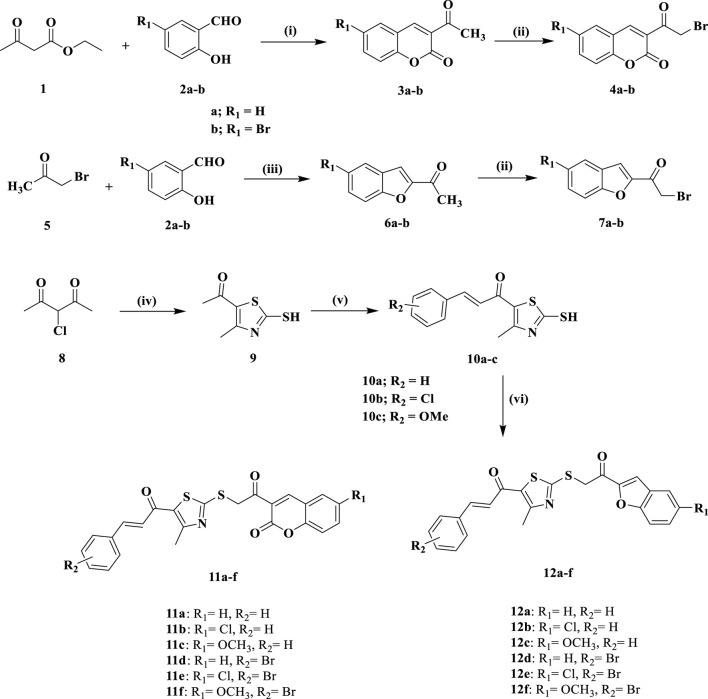
Synthesis of new compounds **11a-f** and **12a-f**.

Scheme 1 highlights the schematic pathway employed for synthesizing the target compounds **11a-f** and **12a-f**. The synthesis was initiated with the condensation of 2-hydroxy benzaldehyde derivatives **2a-b** with ethyl acetoacetate in the presence of piperidine, employing ethanol as the solvent. The reaction was performed at 0°C for 24 h to yield 3-acetyl-2*H*-chromen-2-one derivatives **3a-b** (Sahu et al., 1996). Following this, the bromination of **3a-b** was conducted using *N*-bromosuccinimide (NBS) and *p*-toluene sulfonic acid (PTSA) in acetonitrile under reflux for 12 h, resulting in the formation of 3-(2-bromoacetyl)-2*H*-chromen-2-ones **4a-b** (Kakkar et al., 2018).

Similarly, compounds **7a-b** were synthesized by condensing compounds **2a-b** with bromoacetone **five** in ethanolic KOH at 0°C for 4 h to yield compounds **6a-b** (Chidan Kumar et al., 2015), which were then brominated with a mixture of NBS and PTSA to yield the benzofuran derivatives **7a-b** (Kakkar et al., 2018). The synthesis of chalcone intermediates **10a-c** is depicted in Scheme 1, commencing with the cyclization of 3-chloropentane-2,4-dione (8) into 1-(2-mercapto-4-methylthiazol-5-yl)ethan-1-one (**9**) *via* reacting with ammonia and carbon disulfide in absolute ethanol (Hashem et al., 2024). The acetyl group of the thiazole ring in **9** then participates in a Claisen-Schmidt condensation reaction with various substituted benzaldehydes in ethanol under basic conditions, yielding chalcone derivatives **10a-c** (Hashem et al., 2024). The thiazole chalcones **10a-c** are subjected to *S*-alkylation with 3-(2-bromoacetyl)-2*H*-chromen-2-ones **4a-b** or benzofuran derivatives **7a-b**, utilizing sodium carbonate and sodium iodide in acetone at room temperature for 6 h. This reaction gives the novel compounds **11a-f** and **12a-f** in substantial yields.

Reagents and conditions: (i) Piperidine, EtOH, 0°C, 24 h; (ii) NBS, PTSA.H_2_O, acetonitrile, reflux, 12 h; (iii) KOH, EtOH, 0°C, 4 h; (iv) CS_2_, NH_3_, ethanol, 20°C, 6 h; (v) appropriate aromatic aldehyde, 60% NaOH, EtOH, 0°C, 18 h; (vi) **4a-b**/**7a-b**, Na_2_CO_3_, NaI, acetone, r.t., 5 h.

The structures of the target compounds **11a-f** and **12a-f** were confirmed by ^1^H NMR, ^13^C NMR, and elemental analysis. The ^1^H NMR spectra exhibited characteristic peaks, including a singlet for the methylene group at δ 4.92–4.98 ppm, a singlet for the methyl protons at δ 2.55–2.61 ppm, the chalcone moiety displays distinct resonances, with the α-proton at δ 7.32–7.63 ppm and the β-proton at δ 7.48–7.77 ppm. In some cases, these signals were well-defined, whereas in others, they overlapped with aromatic protons. The aromatic protons corresponding to the coumarin, benzofuran, and benzene rings appeared in their expected region of δ 6.5–8.5 ppm, while the characteristic singlet for the C5 proton of the coumarin moiety was observed at a higher chemical shift (8.73–8.81 ppm). The ^13^C NMR spectra further supported the structural elucidation, revealing distinctive signals for the carbonyl moiety of the chalcone moiety at δ 180–183 ppm, the methylene carbon at δ 38–39 ppm, the methyl carbon at δ 16–19 ppm, and the methoxy carbon at approximately δ 55 ppm.

As a representative example, compound **11f** exhibited well-defined NMR spectral features. In the ^1^H NMR spectrum, the C5 proton of the coumarin core resonated as a singlet at δ 8.73 ppm, whereas the α-proton of the chalcone moiety appeared at δ 7.63 ppm and the β-proton at δ 7.72 ppm, both as doublets. The methylene (-CH_2_-) group was observed as a singlet at δ 4.92 ppm, while the methoxy group exhibited a singlet at δ 3.77 ppm. The methyl group attached to the thiazole ring was also detected as a singlet at δ 2.59 ppm, aligning with its expected chemical shift. The ^13^C NMR spectrum of **11f** further confirmed its structure, displaying a carbonyl signal for the chalcone moiety at δ 193.48 ppm and another carbonyl signal adjacent to the coumarin ring at δ 182.10 ppm. The methoxy carbon was observed at δ 55.94 ppm, the methylene carbon at δ 38.11 ppm, and the methyl carbon at δ 19.03 ppm. These chemical shifts were entirely consistent with the proposed structure, confirming the successful synthesis of **11a-f** and **12a-f**.

### 2.2 Biology

#### 2.2.1 Evaluation of cell viability effect

To assess the viability impact of new targets **11a-f** and **12a-f**, the human mammary gland epithelial (MCF-10A) normal cell line was used. The MTT test was applied to assess the cell viability of **11a-f** and **12a-f** following 4 days of incubation with MCF-10A cells (El-Sherief et al., 2019; Ramadan et al., 2020). Table 1 indicates that none of the analyzed compounds exhibited cytotoxicity since all compounds maintained over 90% cell viability at a concentration of 50 µM.

**TABLE 1:** Cell viability assay and IC_50_ values of compounds **11a-f** and **12a-f** against four cancer cell lines.

Comp	Cell viability %	Antiproliferative activity IC_50_ ± SEM (nM)				
		A-549	MCF-7	Panc-1	HT-29	Average (GI_50_)
**11a**	91	40 ± 2	48 ± 3	44 ± 3	44 ± 3	44
**11b**	92	69 ± 6	74 ± 6	70 ± 6	72 ± 6	71
**11c**	90	52 ± 4	56 ± 4	54 ± 4	57 ± 4	55
**11d**	95	28 ± 1	31 ± 1	30 ± 1	30 ± 1	30
**11e**	91	37 ± 2	43 ± 2	40 ± 2	42 ± 2	41
**11f**	93	25 ± 1	29 ± 1	26 ± 1	28 ± 1	27
**12a**	93	65 ± 5	70 ± 5	66 ± 5	66 ± 5	67
**12b**	90	61 ± 5	68 ± 5	64 ± 5	65 ± 5	65
**12c**	91	33 ± 2	39 ± 2	36 ± 2	37 ± 2	36
**12d**	90	45 ± 3	50 ± 3	48 ± 3	48 ± 3	48
**12e**	94	60 ± 5	64 ± 5	62 ± 5	62 ± 5	62
**12f**	92	55 ± 4	59 ± 4	56 ± 4	56 ± 4	57
**Erlotinib**	ND	30 ± 3	40 ± 3	30 ± 3	30 ± 3	33

#### 2.2.2 Antiproliferative assay

The MTT assay was employed to assess the antiproliferative effects of targets **11a-f** and **12a-f** on four human cancer cell lines: colon cancer (HT-29), pancreatic cancer (Panc-1), lung cancer (A-549), and breast cancer (MCF-7), with erlotinib serving as a reference compound (Mahmoud et al., 2023; Alshammari et al., 2022). Table 1 presents each compound’s median inhibitory concentration (IC_50_) and average IC_50_ (GI_50_) for each compound against the four cancer cell lines.

Results from Table 1 revealed that compounds **11a-f** and **12a-f** exhibited notable antiproliferative efficiency, with GI_50_ values between 27 nM and 71 nM relative to the reference Erlotinib (GI_50_ = 33 nM), and all evaluated compounds demonstrated greater sensitivity towards the lung cancer (A-549) cell line compared to the other cell lines assessed. Furthermore, coumarin-based compounds (**11a-f**) exhibit greater reactivity than benzofuran counterparts (**12a-f**). Compounds **11a**, **11d**, **11e**, **11f**, **12c**, and **12d** exhibited the most potent antiproliferative activity, with GI_50_ values of 44, 30, 41, 27, 36, and 48 nM, respectively, rendering compounds **11d** and **11f** more potent than erlotinib (GI_50_ = 33 nM).

Compound **11f** (R_1_ = 4-OMe, R_2_ = Br, coumarin-based) had the highest efficacy among all synthesized derivatives, demonstrating a GI_50_ value of 27 nM, which is 1.2-fold more active than erlotinib (GI_50_ = 33 nM) against the four tested cancer cell lines.

Compound **11f** exhibited more potency than erlotinib against the four evaluated cancer cell lines. It demonstrated optimal efficacy against lung cancer (A-549) and breast cancer (MCF-7) cell lines. Compound **11f** exhibited IC_50_ values of 25 nM for A-549 and 29 nM for MCF-7, demonstrating 1.2- and 1.4-fold more potency than erlotinib (IC_50_ values = 30 and 40 nM, respectively).

The substitution pattern at the fourth position of the phenyl group in the chalcone moiety (R_1_), along with the type of substitution in the phenyl groups of the coumarin or benzofuran moieties (R_2_), significantly influences the antiproliferative activity of compounds **11a-f** and **12a-f**. For example, compounds **11d** (R_1_ = H, R_2_ = Br, coumarin-based) and **11e** (R_1_ = Cl, R_2_ = Br, coumarin-based), which share identical structural characteristics with compound **11f** but possess different R_1_ substituents, exhibited diminished antiproliferative activity. Compounds **11d** and **11e** had GI_50_ values of 30 and 41 nM, respectively, which are 1.1- and 1.5-fold lower than compound **11f**. The data reveal that the electron-donating group (OMe) is more tolerant of activity than the electron-withdrawing group, with the order of activity (R_1_) being OMe > H > Cl.

Compound **11c** (R_1_ = 4-OMe, R_2_ = H, coumarin-based), which features an unsubstituted coumarin ring, demonstrated a marked decrease in antiproliferative efficacy. Compound **11c** showed a GI_50_ value of 55 nM, demonstrating a twofold inferior potency to that of compound **11f**. This result demonstrates the importance of the bromine atom in the coumarin moiety for antiproliferative action. Moreover, compound **12f** (R_1_ = 4-OMe, R_2_ = Br, benzofuran-based), which shares the same structure as **11f** but incorporates a benzofuran moiety in place of coumarin, exhibited a significant reduction in antiproliferative efficacy. Compound **12f** exhibited a GI_50_ value of 57 nM, indicating it is 2.1-fold less effective than compound **11f**, illustrating that the coumarin moiety is superior for antiproliferative activity than the benzofuran moiety.

A similar principle can be used to compare compounds **11d** (R_1_ = H, R_2_ = Br, coumarin-derived) and **11e** (R_1_ = Cl, R_2_ = Br, coumarin-derived), both of which are coumarin derivatives, to compounds **12d** (R_1_ = H, R_2_ = Br, benzofuran-derived) and **12e** (R_1_ = Cl, R_2_ = Br, benzofuran-derived), both of which are benzofuran derivatives. Compound **12d** showed a GI_50_ value of 48 nM, which was 1.6-fold less effective than the coumarin-based derivative **11d** (GI_50_ = 30 nM), while compound **12e** demonstrated a GI_50_ value of 62 nM, which was 1.5-fold less efficient than its coumarin counterpart, derivative **11e** (GI_50_ = 41 nM).

#### 2.2.3 Evaluation of EGFR inhibitory activity

The most effective antiproliferative compounds, **11a**, **11d**, **11e**, **11f**, and **12c**, were evaluated for their capacity to inhibit EGFR utilizing the EGFR-TK assay (Al-Wahaibi et al., 2022). The findings are presented in Table 2.

**TABLE 2:** IC_50_ values of compounds **11a**, **11d**, **11e**, **11f**, and **12c** against EGFR and VEGFR-2.

Compound	EGFR inhibition IC_50_ ± SEM (nM)	VEGFR-2 inhibition IC_50_ ± SEM (µM)
**11a**	93.00 ± 4	5.20 ± 0.030
**11d**	76.00 ± 3	3.60 ± 0.020
**11e**	89.00 ± 4	4.90 ± 0.030
**11f**	71.00 ± 3	2.90 ± 0.010
**12c**	83.00 ± 3	4.05 ± 0.020
**Erlotinib**	80.00 ± 5	--
**Sorafenib**	--	0.17 ± 0.001

The results of this assay correspond with the antiproliferative assay findings, indicating that compounds **11d** (R_1_ = H, R_2_ = Br, coumarin-based) and **11f** (R_1_ = OMe, R_2_ = Br, coumarin-based) are the most effective derivatives of EGFR inhibitors, exhibiting IC_50_ values of 76 ± 3 and 71 ± 3 nM, respectively, surpassing the potency of the reference drug Erlotinib (IC_50_ = 80 ± 5 nM). Compounds **11d** and **11f** were the most effective derivatives exhibiting antiproliferative effects. Compounds **11e** (R_1_ = Cl, R_2_ = Br, coumarin-based) and **12c** (R_1_ = OMe, R_2_ = H, benzofuran-based) exhibited significant anti-EGFR activity, with IC_50_ values of 89 ± 3 nM and 83 ± 3 nM, respectively. In contrast compound **11a** showed the lowest potency as an EGFR inhibitor, with an IC_50_ value of 93 ± 4 nM, compared to Erlotinib (IC_50_ = 80 nM). The results indicate that compounds **11d** and **11f** have significant antiproliferative activity and may serve as EGFR inhibitors.

#### 2.2.4 Evaluation of VEGFR-2 inhibitory action

Compounds **11a**, **11d**, **11e**, **11f**, and **12c** were evaluated for their capacity to inhibit VEGFR-2, with Sorafenib serving as the control agent (Al-Wahaibi et al., 2024a). The results are displayed as IC_50_ values in Table 2. The findings indicated that the examined compounds had moderate to good VEGFR-2 inhibitory activity, with IC_50_ values between 2.90 and 5.20 µM, compared to Sorafenib, which had an IC_50_ value of 0.17 µM. In all cases, the evaluated compounds exhibit a potency 17-fold inferior to the reference Sorafenib. Compound **11f** (R_1_ = OMe, R_2_ = Br, coumarin-based), the most effective antiproliferative and EGFR inhibitor, also exhibited the highest potency as a VEGFR-2 inhibitor, with an IC_50_ value of 2.90 ± 0.010 µM. Based on the *in vitro* experiments, we infer that compound **11f** exhibits significant antiproliferative activity and functions as a dual inhibitor of EGFR and VEGFR-2, necessitating structural modifications to enhance its efficacy.

### 2.3 Evaluation of antioxidant activity

Antioxidant agents have become essential in medicine due to their extensive preventative and therapeutic usage across many diseases. Free radicals are integral to cancer, cardiovascular diseases, autoimmune illnesses, and age-related issues, prompting novel medical strategies (Goyal et al., 2025). The scavenging of stable free radicals by DPPH (2,2-diphenyl-1-picrylhydrazyl) (El-Sheref et al., 2023) was employed to assess the possible antioxidant capabilities of compounds **11d**, **11f**, and **12c**, with Trolox serving as a reference, Table 3. The assay was performed at three distinct concentrations of the examined compounds (100, 50, and 10 µM).

**TABLE 3:** Antioxidant activity of compounds **11d**, **11f**, and **12c**.

Antioxidant (DPPH radical scavenging activity %)			
Comp	100 µM	50 µM	10 µM
**11d**	92	80	71
**11f**	94	82	73
**12c**	85	73	66
**Trolox**	95	83	78

Compounds **11d** and **11f**, derivatives of coumarin, had substantial antioxidant activity at 10 μM, scavenging DPPH radicals by 71% and 73%, respectively, compared to Trolox (78%). Moreover, compounds **11d** and **11f** exhibited comparable radical scavenging activity to Trolox at concentrations of 100 and 50 μM, respectively (Table 3). Conversely, compound 9, a benzofuran derivative, was identified as the least active derivative regarding antioxidant activity, underscoring the significance of the coumarin moiety for such action. The results suggested that compounds **11d** and **11f** may be considered effective antiproliferative agents with antioxidant properties.

### 2.4 Evaluation of antimicrobial activity

#### 2.4.1 *E. coli* DNA gyrase inhibitory action

A supercoiling experiment was performed to assess the inhibitory efficacy of compounds **11a-f** and **12a-f** against *E. coli* DNA gyrase (Abdel-Aziz et al., 2023). Results are presented as residual activity (RA) of the enzyme at 1 μM of compounds or IC_50_ values for compounds with RA < 50% as presented in Table 4.

**TABLE 4:** IC_50_ values of compounds **11a-f** and **12a-f** against *E. Coli* DNA gyrase.

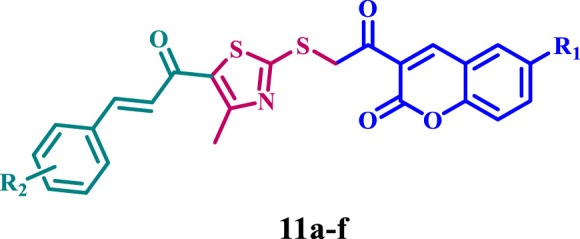		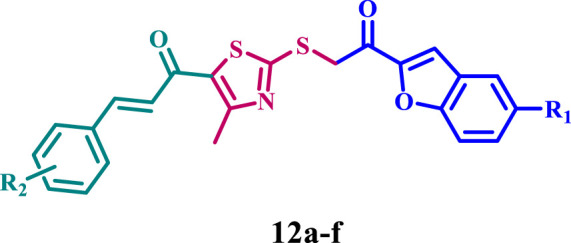	
Compound	R_1_	R_2_	IC_50_ (nM) or RA (%)
			*E. Coli* DNA gyrase
**11a**	H	H	72%
**11b**	Cl	H	182 ± 13
**11c**	OMe	H	68%
**11d**	H	Br	54%
**11e**	Cl	Br	190 ± 13
**11f**	OMe	Br	71%
**12a**	H	H	61%
**12b**	Cl	H	197 ± 12
**12c**	OMe	H	59%
**12d**	H	Br	65%
**12e**	Cl	Br	217 ± 15
**12f**	OMe	Br	208 ± 15
**Novobiocin**	----	----	170 ± 11 nM

Compounds **11a-f** and **12a-f** show notable inhibitory activity against *E. coli* DNA gyrase, with IC_50_ values ranging from 182 to 208 nM, compared to the reference novobiocin, which has an IC_50_ value of 170 nM. The evaluated compounds exhibited lower potency in all cases than the reference novobiocin. Compounds **11b**, **11e**, and **12b** demonstrated the most significant inhibitory activity against *E. coli* DNA gyrase, with IC_50_ values of 182, 190, and 197 nM, respectively. Compound **11b** (R_1_ = Cl, R_2_ = H, coumarin-based) exhibited the highest efficacy as a DNA gyrase inhibitor, with an IC_50_ value of 182 nM, which is 1.1-fold less effective than the reference novobiocin, which has an IC_50_ value of 170 nM. Compound **11e** (R_1_ = Cl, R_2_ = Br, coumarin-based) exhibited the second highest activity as a DNA gyrase inhibitor, with an IC_50_ value of 190 nM, demonstrating equipotency with compound **11b**, so underscoring the significance of halogen atoms in the inhibitory action against DNA gyrase.

Compound **12b**, a benzofuran-based derivative (R_1_ = Cl, R_2_ = H), possesses identical structural characteristics to compound **11b**, but includes a benzofuran moiety instead of a coumarin moiety. It exhibits an IC_50_ value of 197 nM, indicating reduced potency compared to compound **11b** (IC_50_ = 182 nM). The data reaffirmed the significance of the coumarin moiety in the efficacy of these compounds as antiproliferative and antibacterial agents. Compounds **12e** and **12f**, both benzofuran derivatives, exhibited significant inhibitory activity against DNA gyrase, with IC_50_ values of 217 and 208 nM, respectively, approximately 1.2-fold less effective than compound **11b**.

#### 2.4.2 Antibacterial activity

The antibacterial efficacy of compounds **11b**, **11e**, and **12b** was evaluated against Gram-positive bacteria (*S. aureus* and *B. subtilis*) and Gram-negative bacteria (*E. coli* and *P. aeruginosa*). Table 5 presents the MICs (nM) of these compounds against the evaluated bacteria, utilizing ciprofloxacin as the reference drug, determined through a twofold serial dilution approach on a 96-well microtiter plate (Frejat F. O. et al., 2022).

**TABLE 5:** MIC values of compounds **11b**, **11e**, and **12b** against four bacterial species.

Minimum inhibitory concentration (MIC) in nM				
Compound	Bacterial species			
	(G^+^)		(G^−^)	
	*B. subtilis*	*S. aureus*	*E. coli*	*P. aeruginosa*
**11b**	18 ± 1	23 ± 1	42 ± 3	45 ± 3
**11e**	22 ± 1	27 ± 1	48 ± 3	53 ± 3
**12b**	29 ± 1	34 ± 2	62 ± 4	64 ± 4
**Ciprofloxacin**	10 ± 1	30 ± 2	60 ± 4	60 ± 4

Compound **11b** (R_1_ = Cl, R_2_ = H, coumarin-based) had the highest potency among the compounds evaluated, with MIC values of 23, 42, and 45 nM against *S. aureus*, *E. coli*, and *P. aeruginosa*, respectively. It exhibited superior efficacy to ciprofloxacin against the examined species but had a MIC value of 18 nM against *B. subtilis*, which is 1.8-fold less efficient than ciprofloxacin (MIC = 10 nM). Compound **11e** (R_1_ = Cl, R_2_ = Br, coumarin-based) demonstrated the second highest efficacy. The MIC values were similar to those of compound **11b** against *S. aureus* and *E. coli*, as shown in Table 5. Nonetheless, it was 2.2 times less efficacious than ciprofloxacin against *B. subtilis*. Ultimately, the benzofuran-based derivative, compound **12b** (R_1_ = Cl, R_2_ = H), exhibited the lowest potency among the derivatives, demonstrating MIC values inferior to those of ciprofloxacin against all tested species. The observations indicate that compounds **11b** and **11e** are effective antibacterial agents with a broad spectrum of activity against Gram-positive and Gram-negative species, potentially functioning as DNA gyrase inhibitors.

### 2.5 Molecular docking

**FIGURE 3:**
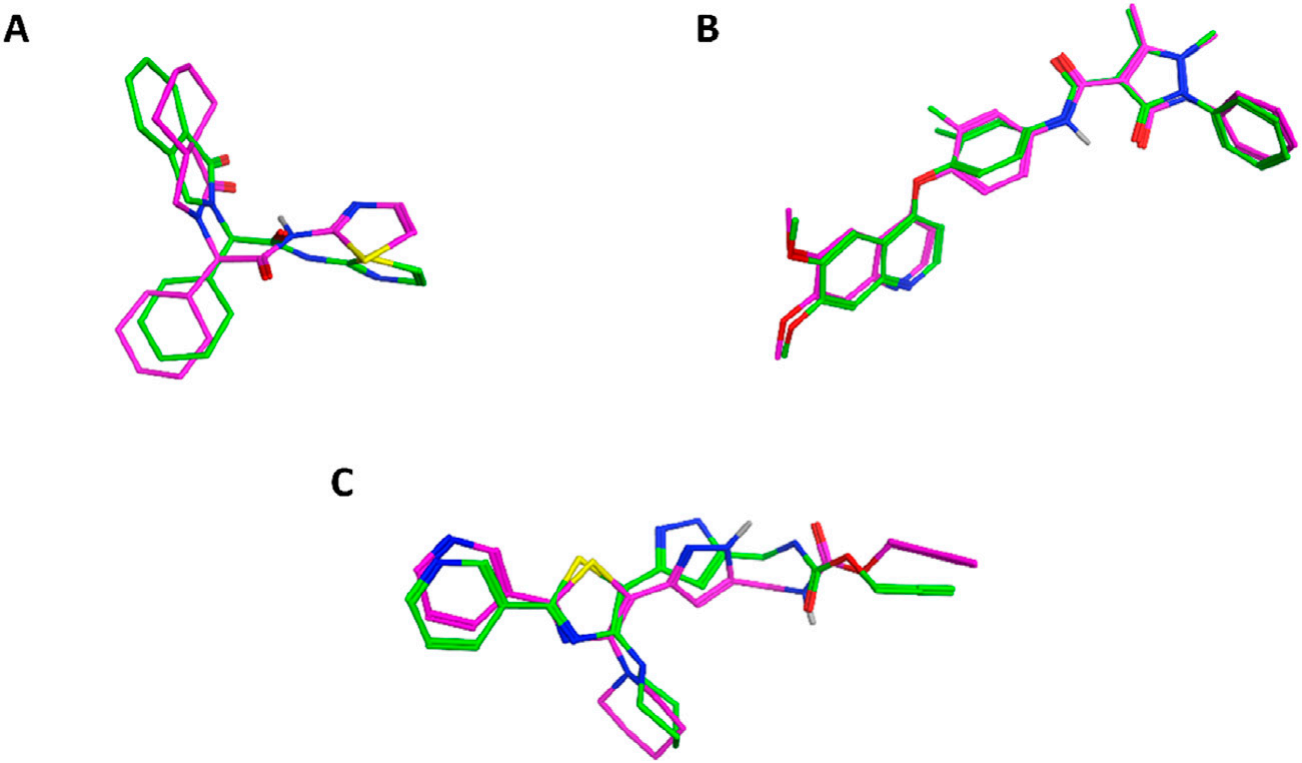
Superimposition of redocked (magenta) and cocrystallized (green) ligands in the active sites of **(A)** EGFR (5D41), **(B)** VEGFR-2 (3U6J), and **(C)** *E. coli* DNA gyrase (3G7E).

The molecular docking studies were conducted to rationalize the *in vitro* potency of the most active compound (**11f**) against three distinct biological targets: EGFR, VEGFR-2, and *E. coli* DNA gyrase. Docking was performed using Auto-Dock Vina (Sharma et al., 2025), and the docking poses were visualized and analyzed with Discovery Studio Visualizer (Rustagi et al., 2025). Initially, docking protocols were validated by redocking the cocrystallized ligands into the corresponding protein active sites to ensure accuracy and reliability. For EGFR, the crystal structure with PDB code 5D41 (Bhanja and Patra, 2025) was utilized. Redocking of the native ligand, EAI001, yielded a binding affinity of −9.3 kcal/mol and an RMSD of 1.1 Å, confirming the validity of the docking conditions. The superimposition between the redocked and cocrystallized ligand is depicted in Figure 3.

**FIGURE 4:**
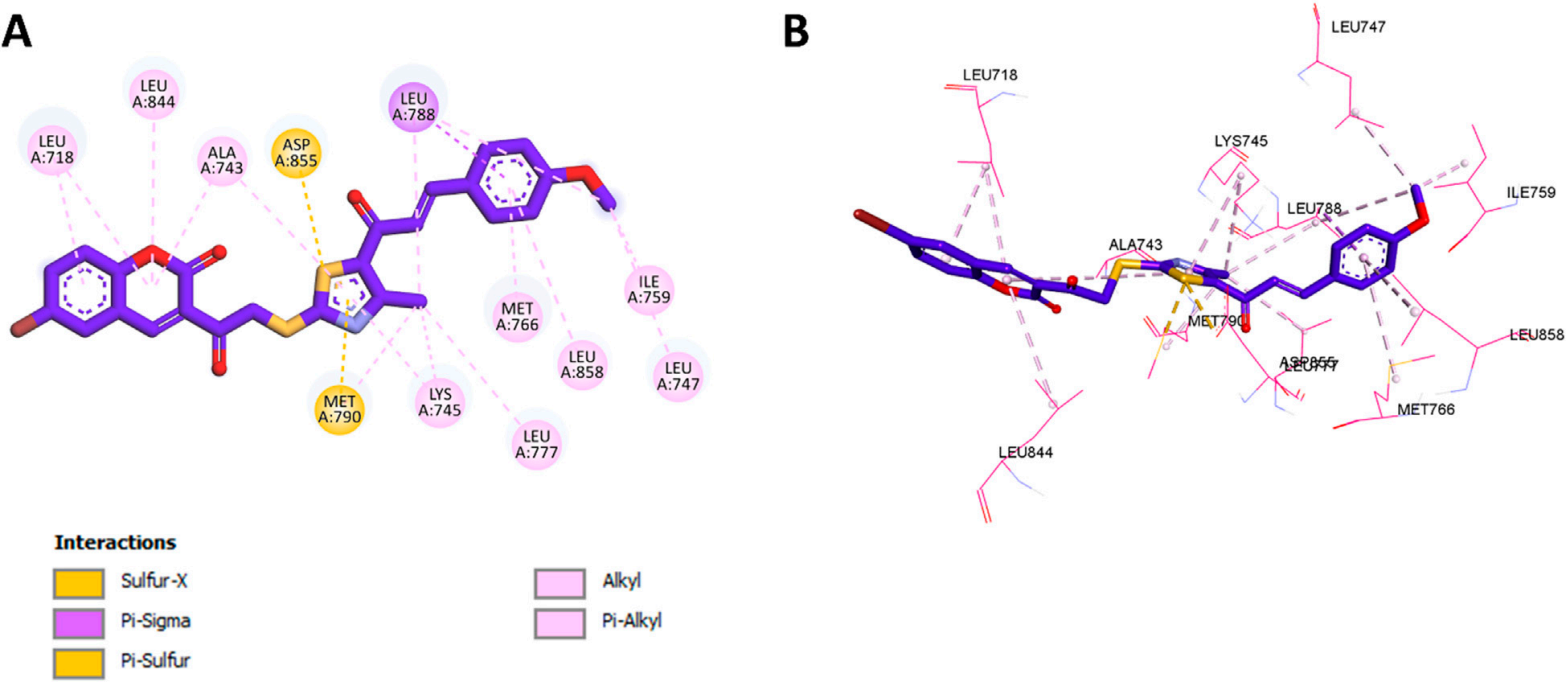
**(A)** 2D interaction diagram and **(B)** 3D binding mode of **11f** within the allosteric site of EGFR.

Subsequently, docking of compound **11f** into the allosteric site of EGFR, previously identified as the binding pocket for EAI001, demonstrated a binding affinity of −8.5 kcal/mol. Detailed interaction analysis revealed that the coumarin ring of **11f** engaged in significant hydrophobic interactions with Leu718, Leu844, and Ala743. Additionally, the thiazole moiety formed hydrophobic contacts with Ala743 and Lys745, alongside a pi-sulfur interaction with Met790 and a sulfur-X interaction with Asp855. The benzene ring of **11f** further stabilized the binding through hydrophobic interactions with Met766, Leu858, and Leu788. Notably, the methoxy group appeared to contribute to the compound’s enhanced potency by participating in hydrophobic interactions with Leu747, Ile759, and Leu788, potentially improving binding affinity compared to other derivatives. A summary of these interactions is presented in Figure 4.

**FIGURE 5:**
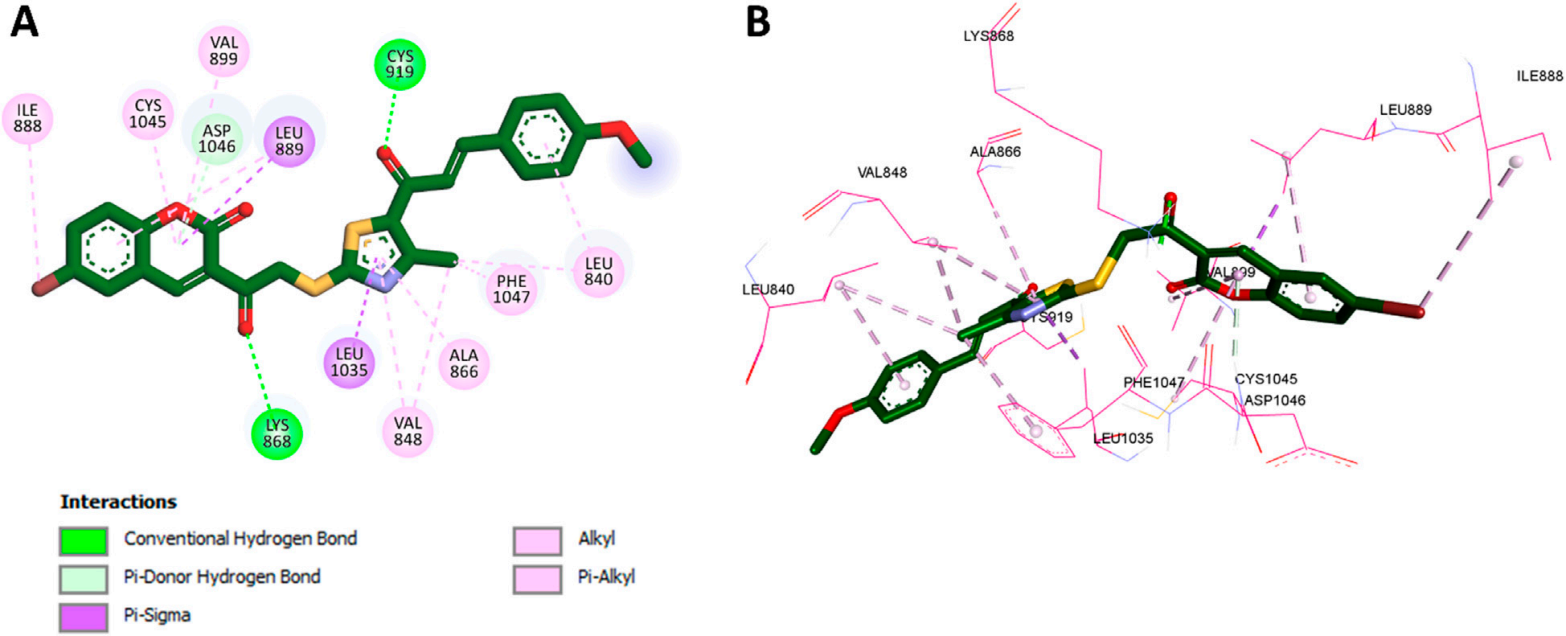
**(A)** 2D interaction diagram and **(B)** 3D binding mode of **11f** within the VEGFR-2 ATP-binding site.

For VEGFR-2, docking was conducted using the crystal structure with PDB code 3U6J (Reang et al., 2023), which represents the ATP-binding site of the kinase. Validation through redocking of the cocrystallized ligand yielded a binding affinity of −7.2 kcal/mol and an RMSD of 0.512 Å, confirming the accuracy of the docking protocol. The superimposition of the redocked and cocrystallized ligand is also shown in Figure 3. Docking of compound **11f** into VEGFR-2 produced a binding affinity of −8.9 kcal/mol, with several notable interactions observed, as presented in Figure 5. The coumarin moiety of **11f** exhibited hydrophobic interactions with Leu889, Val899, and Cys1045 and formed a pi-donor hydrogen bond with Asp1046. Additionally, the bromine substituent on the coumarin ring engaged in hydrophobic interaction with Ile888. The carbonyl groups of **11f** formed classical hydrogen bonds with key residues Cys919 and Lys868, further stabilizing the complex. Moreover, the thiazole ring contributed to the binding through hydrophobic interactions with Leu1035, Val848, Ala866, Phe1047, and Leu840.

**FIGURE 6:**
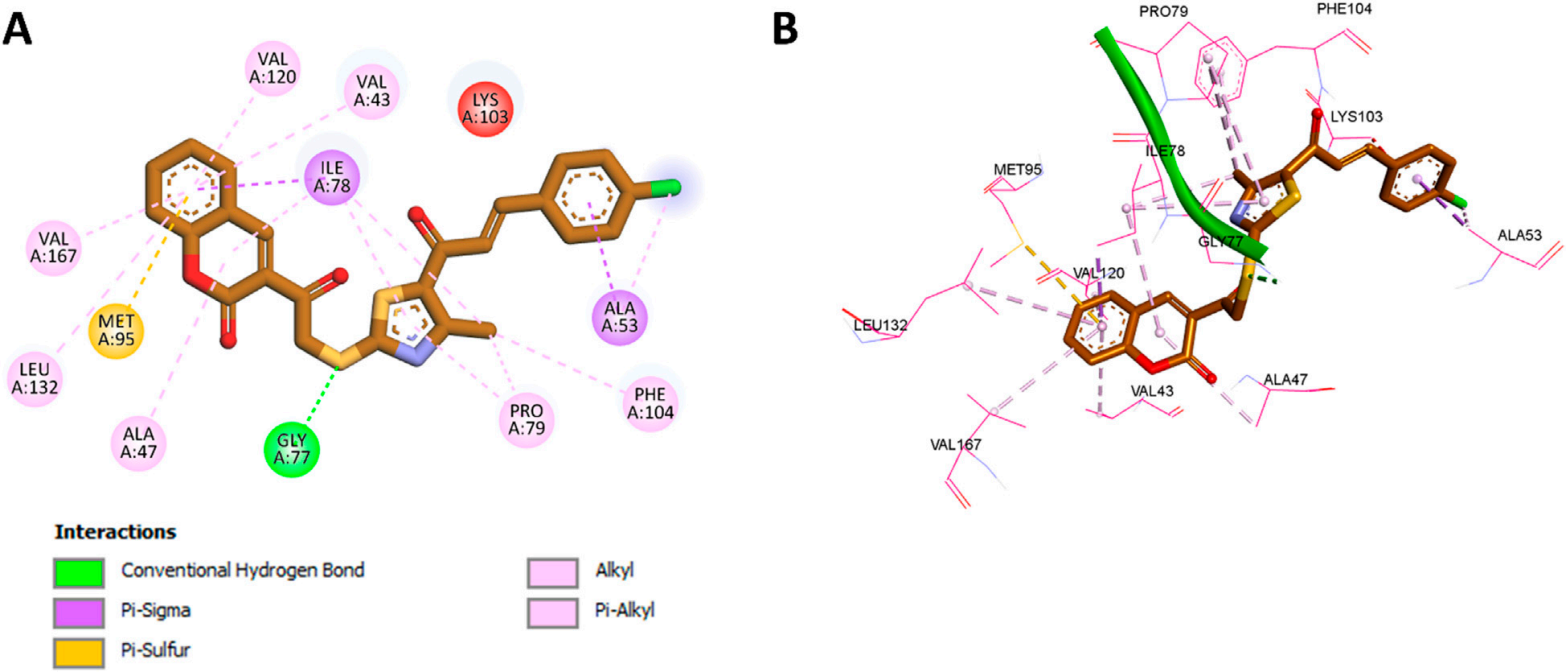
**(A)** 2D interaction diagram and **(B)** 3D binding mode of **11b** within the ATP-binding site of the GyrB subunit.

For *E. coli* DNA gyrase, docking studies were performed using the crystal structure with PDB code 3G7E (Sofi et al., 2025), which represents the ATP-binding site of the GyrB subunit. Redocking of the native ligand resulted in a binding affinity of −6.7 kcal/mol and an RMSD of 1.126 Å, confirming the suitability of the docking settings. The superimposition of the redocked and cocrystallized ligand is included in Figure 3. Docking of compound **11b**, which exhibited the highest *in vitro* potency against DNA gyrase, resulted in a binding affinity of −9.3 kcal/mol. The coumarin moiety of **11b** was found to establish extensive hydrophobic interactions with Val120, Val43, Ile78, Val167, Leu132, and Ala47, along with a pi-sulfur interaction with Met95. The thiazole ring of **11b** further contributed hydrophobic interactions with Ile78 and Pro79. Notably, the sulfur atom adjacent to the thiazole moiety formed a classical hydrogen bond with Gly77. In addition, both the phenyl ring and the chlorine substituent in **11b** engaged in hydrophobic interactions with Ala53. A comprehensive summary of these interactions is depicted in Figure 6.

## 3 Conclusion

In this study, we designed, synthesized, and biologically evaluated a novel series of thiazole-based derivatives (**11a-f** and **12a-f**) as multi-targeted inhibitors with potent antiproliferative, antioxidant, and antibacterial activities. Compounds **11d** and **11f** exhibited the strongest antiproliferative effects (GI_50_ = 30 and 27 nM, respectively), surpassing Erlotinib, through dual EGFR/VEGFR-2 inhibition. Structure-activity relationship analysis highlighted the critical role of the coumarin moiety and electron-donating substituents in enhancing activity. Additionally, **11d** and **11f** demonstrated robust antioxidant potential. This dual antiproliferative and antioxidant profile is particularly valuable, as oxidative stress plays a significant role in cancer pathogenesis and progression. Furthermore, **11b**, **11e**, and **12b** exhibited potent antibacterial activity, inhibiting *E. coli* DNA gyrase and displaying broad-spectrum efficacy. The multi-target profile of these derivatives underscores their potential as versatile therapeutic agents against cancer and bacterial infections. Further studies are required to assess their *in vivo* efficacy, pharmacokinetics, and toxicity. Future research will focus on optimizing VEGFR-2 inhibition while retaining the compounds’ anticancer, antioxidant, and antibacterial properties.

In summary, these thiazole-based derivatives represent a promising class of multi-targeted inhibitors with significant potential for the development of new therapeutic agents addressing both cancer and bacterial infections, two major global health challenges. The dual EGFR/VEGFR-2 inhibition mechanism, coupled with antioxidant and antibacterial properties, positions these compounds as versatile candidates for further development in medicinal chemistry and drug discovery pipelines.

## 4 Experimental

### 4.1 Chemistry

General Details: Refer to Appendix A.

#### 4.1.1 General procedure for the synthesis of target compounds (11a-f) and (12a-f)

A mixture of thiazole chalcones **10a-c** (1 mmol) and the corresponding coumarins **4a-b** or benzofurans **7a-b** (1 mmol) in acetone containing sodium carbonate (1.5 mmol, 0.159 g) and sodium iodide (2 mmol, 0.3 g) was stirred at room temperature for 5 h. After the reaction was complete, the mixture was refrigerated overnight. The precipitate was filtered, washed with distilled water, and recrystallized using ethanol.

##### 4.1.1.1 (*E*)-3-(2-((5-Cinnamoyl-4-methylthiazol-2-yl)thio)acetyl)-2*H*-chromen-2-one (11a)

Yellow powder; 0.312 g, 70% yield; m.p. 215°C–217°C; ^1^H NMR (500 MHz, DMSO-*d*_6_) δ 8.81 (s, 1H, coumarin-C_4_-H), 8.02–7.98 (m, 2H, Ar-H), 7.76 (d, *J* = 15.3 Hz, 1H, chalcone-β C=H), 7.66–7.58 (m, 5H, Ar-H & chalcone-α C=H), 7.39–7.31 (m, 3H, Ar-H), 4.95 (s, 2H, CH_2_), 2.60 (s, 3H, CH_3_); ^13^C NMR (100 MHz, DMSO-*d*_6_) δ 190.33, 181.78, 166.60, 164.28, 159.32, 155.06, 145.08, 138.06, 137.05, 134.06, 132.07, 130.84, 130.09, 126.06, 125.11, 124.38, 122.83, 120.13, 119.32, 116.85, 38.45, 18.81. Anal. Calc. (%) for C_24_H_17_NO_4_S_2_: C, 64.41; H, 3.83; N, 3.13. Found: C, 64.33; H, 3.87; N, 3.17.

##### 4.1.1.2 (*E*)-3-(2-((5-(3-(4-Chlorophenyl)acryloyl)-4-methylthiazol-2-yl)thio)acetyl)-2*H*-chromen-2-one (11b)

Yellow powder; 0.366 g, 76% yield; m.p. 222°C–223°C; ^1^H NMR (500 MHz, DMSO-*d*_6_) δ 8.81 (s, 1H, coumarin-C_4_-H), 8.02–7.97 (m, 2H, Ar-H), 7.72 (d, *J* = 15.4 Hz, 1H, chalcone-β C=H), 7.66–7.59 (m, 3H, Ar-H & chalcone-α C=H), 7.54 (d, *J* = 8.2 Hz, 2H, Ar-H), 7.43 (d, *J* = 8.4 Hz, 2H, Ar-H), 4.95 (s, 2H, CH_2_), 2.61 (s, 3H, CH_3_); ^13^C NMR (100 MHz, DMSO-*d*_6_) δ 191.02, 180.88, 166.56, 163.87, 157.18, 153.19, 147.11, 141.15, 137.16, 133.47, 131.68, 130.30, 129.04, 126.04, 125.33, 124.62, 121.89, 120.85, 119.44, 116.97, 38.68, 19.25. Anal. Calc. (%) for C_24_H_16_ClNO_4_S_2_: C, 59.81; H, 3.35; N, 2.91. Found: C, 59.74; H, 3.39; N, 2.84.

##### 4.1.1.3 (*E*)-3-(2-((5-(3-(4-Methoxyphenyl)acryloyl)-4-methylthiazol-2-yl)thio) acetyl)-2*H*-chromen-2-one (11c)

Yellow powder; 0.329 g, 69% yield; m.p. 229°C–231°C; ^1^H NMR (500 MHz, DMSO-*d*_6_) δ 8.80 (s, 1H, coumarin-C_4_-H), 8.01–7.98 (m, 2H, Ar-H), 7.72 (d, *J* = 15.3 Hz, 1H, chalcone-β C=H), 7.66–7.62 (m, 2H, Ar-H & chalcone-α C=H), 7.61–7.59 (m, 2H, Ar-H), 7.58 (s, 1H, Ar-H), 6.92 (d, *J* = 8.4 Hz, 2H, Ar-H), 4.94 (s, 2H, CH_2_), 3.79 (s, 3H, OCH_3_), 2.58 (s, 3H, thiazole-CH_3_); ^13^C NMR (100 MHz, DMSO-*d*_6_) δ 192.16, 180.16, 166.18, 160.61, 156.76, 153.26, 150.68, 143.56, 138.33, 137.03, 134.17, 131.25, 130.55, 127.31, 125.69, 124.07, 123.11, 122.19, 119.22, 118.58, 55.92, 38.43, 19.02. Anal. Calc. (%) for C_25_H_19_NO_5_S_2_: C, 62.88; H, 4.01; N, 2.93. Found: C, 62.76; H, 4.13; N, 3.87.

##### 4.1.1.4 (*E*)-6-Bromo-3-(2-((5-cinnamoyl-4-methylthiazol-2-yl)thio)acetyl)-2*H*-chromen-2-one (11d)

Yellow powder; 0.405 g, 77% yield; m.p. 255°C–257°C; ^1^H NMR (500 MHz, DMSO-*d*_6_) δ 8.73 (s, 1H, coumarin-C_4_-H), 8.22 (s, 1H, Ar-H), 7.97 (d, *J* = 8.9 Hz, 1H, Ar-H), 7.77–7.74 (m, 2H, Ar-H & chalcone-βC = H), 7.63 (d, *J* = 15.4 Hz, 1H, chalcone-αC = H), 7.59 (dd, *J* = 6.9, 2.4 Hz, 2H, Ar-H), 7.39–7.31 (m, 3H, Ar-H), 4.93 (s, 2H, CH_2_), 2.59 (s, 3H, CH_3_); ^13^C NMR (100 MHz, DMSO-*d*_6_) δ 190.55, 180.84, 169.14, 164.58, 158.37, 151.95, 145.48, 144.16, 140.91, 140.33, 137.05, 134.46, 132.17, 129.87, 129.29, 128.27, 124.76, 120.54, 119.24, 116.94, 37.18, 18.73. Anal. Calc. (%) for C_24_H_16_BrNO_4_S_2_: C, 54.67; H, 3.06; N, 2.66. Found: C, 54.58; H, 3.15; N, 2.57.

##### 4.1.1.5 (*E*)-6-Bromo-3-(2-((5-(3-(4-chlorophenyl)acryloyl)-4-methylthiazol-2-yl)thio)acetyl)-2*H*-chromen-2-one (11e)

Yellow powder; 0.398 g, 71% yield; m.p. 236°C–237°C; ^1^H NMR (500 MHz, DMSO-*d*_6_) δ 8.73 (s, 1H, coumarin-C_4_-H), 8.22 (d, *J* = 2.3 Hz, 1H, Ar-H), 7.90 (dd, *J* = 8.9, 2.4 Hz, 1H, Ar-H), 7.80 (d, *J* = 8.5 Hz, 2H, Ar-H), 7.62 (d, *J* = 15.5 Hz, 1H, chalcone-β C=H), 7.47 (dd, *J* = 8.7, 2.5 Hz, 3H, Ar-H), 7.34 (d, *J* = 15.5 Hz, 1H, chalcone-α C=H), 4.93 (s, 2H, CH_2_), 2.59 (s, 3H, CH_3_); ^13^C NMR (100 MHz, DMSO-*d*_6_) δ 190.57, 181.22, 171.59, 160.41, 155.56, 148.87, 144.02, 138.80, 135.91, 134.31, 132.84, 132.10, 131.13, 130.19, 128.72, 124.24, 121.66, 118.65, 117.19, 113.43, 37.84, 18.14. Anal. Calc. (%) for C_24_H_15_BrClNO_4_S_2_: C, 51.40; H, 2.70; N, 2.50. Found: C, 51.28; H, 2.76; N, 2.44.

##### 4.1.1.6 (*E*)-6-Bromo-3-(2-((5-(3-(4-methoxyphenyl)acryloyl)-4-methylthiazol-2-yl)thio)acetyl)-2*H*-chromen-2-one (11f)

Yellow powder; 0.456 g, 82% yield; m.p. 232°C–235°C; ^1^H NMR (500 MHz, DMSO-*d*_6_) δ 8.73 (s, 1H, coumarin-C_4_-H), 8.10 (d, *J* = 2.4 Hz, 1H, Ar-H), 7.97 (d, *J* = 8.9 Hz, 1H, Ar-H), 7.75 (dd, *J* = 8.9, 2.5 Hz, 1H, Ar-H), 7.72 (d, *J* = 15.3 Hz, 1H, chalcone-β C=H), 7.63 (d, *J* = 15.4 Hz, 1H, chalcone-α C=H), 7.58 (d, *J* = 8.0 Hz, 2H, Ar-H), 6.93 (d, *J* = 8.7 Hz, 2H, Ar-H), 4.92 (s, 2H, CH_2_), 3.77 (s, 3H, OCH_3_), 2.59 (s, 3H, thiazole-CH_3_); ^13^C NMR (100 MHz, DMSO-*d*_6_) δ 193.48, 182.10, 166.58, 159.74, 152.98, 144.81, 143.56, 139.65, 139.26, 136.44, 135.40, 132.16, 130.56, 129.58, 127.68, 125.70, 124.10, 122.17, 119.90, 114.66, 55.94, 38.11, 19.03. Anal. Calc. (%) for C_25_H_18_BrNO_4_S_2_: C, 53.96; H, 3.26; N, 2.52. Found: C, 53.84; H, 3.37; N, 2.57.

##### 4.1.1.7 (*E*)-1-(2-((2-(Benzofuran-2-yl)-2-oxoethyl)thio)-4-methylthiazol-5-yl)-3-phenylprop-2-en-1-one (12a)

Yellow powder; 0.281 g, 67% yield; m.p. 219°C–221°C; ^1^H NMR (500 MHz, DMSO-*d*_6_) δ 8.12 (s, 1H, Ar-H), 7.73 (d, *J* = 15.3 Hz, 1H, chalcone-β C=H), 7.68 (d, *J* = 8.0 Hz, 1H, Ar-H), 7.62 (d, *J* = 8.4 Hz, 2H, Ar-H), 7.53 (d, *J* = 8.0 Hz, 1H, Ar-H), 7.48 (d, *J* = 15.3 Hz, 1H, chalcone-α C=H), 7.48–7.43 (m, 5H, Ar-H), 4.98 (s, 2H, CH_2_), 2.55 (s, 3H, CH_3_); ^13^C NMR (100 MHz, DMSO-*d*_6_) δ 183.56, 179.86, 167.21, 162.02, 151.27, 144.53, 142.87, 140.32, 138.59, 136.45, 135.09, 133.22, 130.88, 129.63, 127.97, 126.08, 124.40, 119.92, 118.90, 38.49, 16.74. Anal. Calc. (%) for C_23_H_17_NO_3_S_2_: C, 65.58; H, 4.08; N, 3.34. Found: C, 65.66; H, 4.02; N, 3.29.

##### 4.1.1.8 (*E*)-1-(2-((2-(Benzofuran-2-yl)-2-oxoethyl)thio)-4-methylthiazol-5-yl)-3-(4-chlorophenyl)prop-2-en-1-one (12b)

Yellow powder; 0.349 g, 77% yield; m.p. 215°C–216°C; ^1^H NMR (500 MHz, DMSO-*d*_6_) δ 8.11 (s, 1H, Ar-H), 7.85 (d, *J* = 7.8 Hz, 1H, Ar-H), 7.78 (d, *J* = 8.6 Hz, 2H, Ar-H), 7.72 (d, *J* = 8.4 Hz, 1H, Ar-H), 7.61 (d, *J* = 15.5 Hz, 1H, chalcone-β C=H), 7.54 (t, *J* = 7.2 Hz, 1H, Ar-H), 7.46 (d, *J* = 8.5 Hz, 2H, Ar-H), 7.36 (t, *J* = 7.2 Hz, 1H, Ar-H), 7.32 (d, *J* = 15.5 Hz, 1H, chalcone-α C=H), 4.97 (s, 2H, CH_2_), 2.54 (s, 3H, CH_3_); ^13^C NMR (100 MHz, DMSO-*d*_6_) δ 183.41, 181.80, 168.48, 158.39, 156.21, 150.98, 142.51, 136.05, 133.48, 132.49, 131.18, 129.55, 127.29, 125.37, 124.75, 123.78, 117.27, 115.62, 112.76, 37.84, 18.73. Anal. Calc. (%) for C_23_H_16_ClNO_3_S_2_: C, 60.85; H, 3.55; N, 3.09. Found: C, 60.94; H, 3.67; N, 3.13.

##### 4.1.1.9 (*E*)-1-(2-((2-(Benzofuran-2-yl)-2-oxoethyl)thio)-4-methylthiazol-5-yl)-3-(4-methoxyphenyl)prop-2-en-1-one (12c)

Yellow powder; 0.386 g, 86% yield; m.p. 227°C–229°C; ^1^H NMR (500 MHz, DMSO-*d*_6_) δ 8.11 (s, 1H, Ar-H), 7.71 (d, *J* = 9.7 Hz, 1H, Ar-H), 7.59 (d, *J* = 8.6 Hz, 2H, Ar-H), 7.52 (d, *J* = 7.8 Hz, 1H, Ar-H), 7.48 (d, *J* = 15.9 Hz, 1H, chalcone-β C=H), 7.44–7.38 (m, 2H, Ar-H & chalcone-α C=H), 7.30–7.25 (m, 1H, Ar-H), 6.93 (d, *J* = 8.7 Hz, 2H, Ar-H), 4.96 (s, 2H, CH_2_), 3.76 (s, 3H, OCH_3_), 2.54 (s, 3H, thiazole-CH_3_); ^13^C NMR (100 MHz, DMSO-*d*_6_) δ 184.09, 181.77, 169.79, 165.26, 158.74, 154.58, 152.29, 151.26, 142.90, 142.21, 137.02, 130.53, 129.51, 127.97, 124.08, 123.41, 119.47, 114.68, 112.75, 55.92, 37.55, 16.45. Anal. Calc. (%) for C_24_H_19_NO_4_S_2_: C, 64.12; H, 4.26; N, 3.12. Found: C, 64.19; H, 4.33; N, 3.04.

##### 4.1.1.10 (*E*)-1-(2-((2-(5-Bromobenzofuran-2-yl)-2-oxoethyl)thio)-4-methylthiazol-5-yl)-3-phenylprop-2-en-1-one (12d)

Yellow powder; 0.438 g, 88% yield; m.p. 245°C–246°C; ^1^H NMR (500 MHz, DMSO-*d*_6_) δ 7.94 (t, *J* = 2.0 Hz, 1H, Ar-H), 7.84 (d, *J* = 2.1 Hz, 1H, Ar-H), 7.72 (d, *J* = 15.9 Hz, 1H, chalcone-β C=H), 7.60–7.54 (m, 4H, Ar-H), 7.47 (d, *J* = 15.9 Hz, 1H, chalcone-α C=H), 7.39–7.31 (m, 3H, Ar-H), 4.97 (s, 2H, CH_2_), 2.54 (s, 3H, CH_3_); ^13^C NMR (100 MHz, DMSO-*d*_6_) δ 183.71, 181.49, 164.94, 163.59, 159.07, 155.83, 154.22, 150.02, 141.96, 138.34, 131.86, 130.26, 128.93, 127.03, 126.06, 119.21, 117.88, 116.64, 112.34, 37.16, 16.72. Anal. Calc. (%) for C_23_H_16_BrNO_3_S_2_: C, 55.43; H, 3.24; N, 2.81. Found: C, 55.39; H, 3.27; N, 2.79.

##### 4.1.1.11 (*E*)-1-(2-((2-(5-bromobenzofuran-2-yl)-2-oxoethyl)thio)-4-methylthiazol-5-yl)-3-(4-chlorophenyl)prop-2-en-1-one (12e)

Yellow powder; 0.362 g, 68% yield; m.p. 251°C–252°C; ^1^H NMR (500 MHz, DMSO-*d*_6_) δ 8.10 (d, *J* = 1.9 Hz, 1H, Ar-H), 8.05 (s, 1H, Ar-H), 7.79 (d, *J* = 8.5 Hz, 2H, Ar-H), 7.74 (d, *J* = 8.9 Hz, 1H, Ar-H), 7.69 (dd, *J* = 8.9, 2.0 Hz, 1H, Ar-H), 7.62 (d, *J* = 15.5 Hz, 1H, chalcone-β C=H), 7.47 (d, *J* = 8.5 Hz, 2H, Ar-H), 7.33 (d, *J* = 15.5 Hz, 1H, chalcone-α C=H), 4.97 (s, 2H, CH_2_), 2.54 (s, 3H, CH_3_); ^13^C NMR (100 MHz, DMSO-*d*_6_) δ 183.09, 179.21, 164.94, 157.79, 155.17, 153.90, 148.03, 143.85, 140.30, 139.32, 138.33, 133.47, 131.85, 127.02, 126.13, 124.75, 123.76, 121.50, 116.94, 37.57, 16.44. Anal. Calc. (%) for C_23_H_15_BrClNO_3_S_2_: C, 51.84; H, 2.84; N, 2.63. Found: C, 51.91; H, 2.89; N, 2.59.

##### 4.1.1.12 (*E*)-1-(2-((2-(5-Bromobenzofuran-2-yl)-2-oxoethyl)thio)-4-methylthiazol-5-yl)-3-(4-methoxyphenyl)prop-2-en-1-one (12f)

Yellow powder; 0.417 g, 79% yield; m.p. 238°C–239°C; ^1^H NMR (500 MHz, DMSO-*d*_6_) δ 7.94 (t, *J* = 2.4 Hz, 1H, Ar-H), 7.84 (d, *J* = 2.1 Hz, 1H, Ar-H), 7.58 (d, *J* = 8.6 Hz, 3H, Ar-H), 7.55 (dd, *J* = 8.5, 2.1 Hz, 1H, Ar-H), 7.48 (d, *J* = 15.9 Hz, 1H, chalcone-β C=H), 7.41 (d, *J* = 15.9 Hz, 1H, chalcone-α C=H), 6.93 (d, *J* = 8.7 Hz, 2H, Ar-H), 4.96 (s, 2H, CH_2_), 3.76 (s, 3H, OCH_3_), 2.58 (s, 3H, thiazole-CH_3_); ^13^C NMR (100 MHz, DMSO-*d*_6_) δ 183.71, 181.49, 165.27, 157.82, 156.21, 146.82, 138.04, 136.73, 134.13, 133.17, 130.25, 129.60, 128.26, 127.31, 123.42, 122.18, 119.89, 119.22, 117.25, 55.99, 37.84, 16.14. Anal. Calc. (%) for C_24_H_18_BrNO_4_S_2_: C, 54.55; H, 3.43; N, 2.65. Found: C, 54.64; H, 3.40; N, 2.67.

### 4.2 Biology

#### 4.2.1 Assay for cell viability

The viability effects of **11a-f** and **12a-f** on the human mammary gland epithelial (MCF-10A) normal cell line were evaluated using the MTT test (El-Sherief et al., 2019; Ramadan et al., 2020). Refer to Appendix A for additional information.

#### 4.2.2 Antiproliferative assay

The MTT assay was employed to assess the antiproliferative efficacy of **11a-f** and **12a-f** against four human cancer cell lines, utilizing Erlotinib as a reference control (Mahmoud et al., 2023; Alshammari et al., 2022). Dose-response assays determined the IC_50_ values for the novel compounds. We derived the reported data from a minimum of two separate experiments, each consisting of three repetitions per concentration. Appendix A provides experimental details.

#### 4.2.3 Assay for EGFR inhibitory action

The most effective antiproliferative compounds, **11a**, **11d**, **11e**, **11f**, and **12c**, were evaluated for their capacity to inhibit EGFR utilizing the EGFR-TK assay (Al-Wahaibi et al., 2022). Refer to Appendix A for more details.

#### 4.2.4 Assay for VEGFR-2 inhibitory action

Compounds **11a**, **11d**, **11e**, **11f**, and **12c** were assessed for their ability to inhibit VEGFR-2, using sorafenib as the control agent (Al-Wahaibi et al., 2024a). The outcomes are presented as IC_50_ values. Appendix A outlines more experimental details.

#### 4.2.5 Antioxidant assay

The scavenging of stable free radicals by DPPH (El-Sheref et al., 2023) was employed to assess the antioxidant activities of compounds **11d**, **11f**, and **12c**, with Trolox serving as a reference. The assay was performed at three different concentrations of the examined compounds (100, 50, and 10 µM). Appendix A contains more details.

#### 4.2.6 DNA gyrase inhibitory assay

A supercoiling experiment was performed to assess the inhibitory efficacy of compounds **11a-f** and **12a-f** against *E. coli* DNA gyrase (Abdel-Aziz et al., 2023). Results are presented as RA of the enzyme at 1 μM of compounds or IC_50_ values for compounds with RA < 50%. Refer to Appendix A for more information.

#### 4.2.7 Antibacterial assay and MIC calculations

The antibacterial efficacy of compounds **11b**, **11e**, and **12b** was evaluated against Gram-positive bacteria (*S. aureus* and *B. subtilis*) and Gram-negative bacteria (*E. coli* and *P. aeruginosa*). The MICs (nM) of the tested compounds against the evaluated bacteria were determined through a twofold serial dilution approach on a 96-well microtiter plate (Frejat F. O. et al., 2022). See Appendix A for more details.

## Data Availability

The original contributions presented in the study are included in the article/Supplementary Material, further inquiries can be directed to the corresponding authors.
