# Supplementary material for: Design, synthesis, and biological investigation of new thiazole-based derivatives as multi-targeted inhibitors endowed with antiproliferative, antioxidant, and antibacterial properties
Source: Front Chem. 2025 May 6;13:1595997. doi: 10.3389/fchem.2025.1595997 (PMC12088962; doi:10.3389/fchem.2025.1595997)
Supplement: Supplementary file 1 [file DataSheet1.pdf]

Design, synthesis, and biological investigation of new thiazole-based derivatives as multi-targeted inhibitors endowed with antiproliferative, antioxidant, and antibacterial properties

Hesham A.M. Gomaa[1*], Asmaa M. Atta[2], Mohamed E. Shaker[1], Sami I. Alzarea[1], Tariq G. Alsahli[1], Eid Alatwi[1], Arafa Musa[3], Fatma A. M. Mohamed[4], Hayat Ali Alzahrani[5], Aliaa M. Mohassab[6], Alshaimaa Abdelmoez[7], Stefan Bräse[8*], Bahaa G. M. Youssif[7*]

[1]Department of Pharmacology, College of Pharmacy, Jouf University, Sakaka 72388, Saudi Arabia; [2]Pharmaceutical Chemistry Department, Faculty of Pharmacy, Badr University in Cairo (BUC), Badr City, Cairo 11829, Egypt; [3]Department of Pharmacognosy, College of Pharmacy, Jouf University, Sakaka, Aljouf 72341, Saudi Arabia; [4]Department of Clinical Laboratory Sciences, College of Applied Medical Sciences at Al-Qurayyat, Jouf University, Al-Qurayyat 77454, Saudi Arabia; [5]Applied Medical Science College, Medical Laboratory Technology Department, Northern Border University, Arar, Saudi Arabia, [6]Department of Medicinal Chemistry, Faculty of Pharmacy, Minia University, Minia 61519, Egypt; [7]Institute of Biological and Chemical Systems, IBCS-FMS, Karlsruhe Institute of Technology, 76131 Karlsruhe, Germany [8]Department of Pharmaceutical Organic Chemistry, Faculty of Pharmacy, Assiut University, Assiut-71526, Egypt.

**To whom correspondence should be addressed:*

Hesham A.M. Gomaa, Ph.D. Department of Pharmacology, College of Pharmacy, Jouf University, Sakaka 72388, Saudi Arabia.

E-mail address: hasoliman@ju.edu.sa

Bahaa G. M. Youssif, Ph.D. Pharmaceutical Organic Chemistry Department, Faculty of Pharmacy, Assiut University, Assiut 71526, Egypt.

Tel.: +201044353895; E-mail address: bgyoussif2@gmail.com

Stefan Bräse

Institute of Biological and Chemical Systems, IBCS-FMS, Karlsruhe Institute of Technology, 76131 Karlsruhe, Germany. E-mail: braese@kit.edu

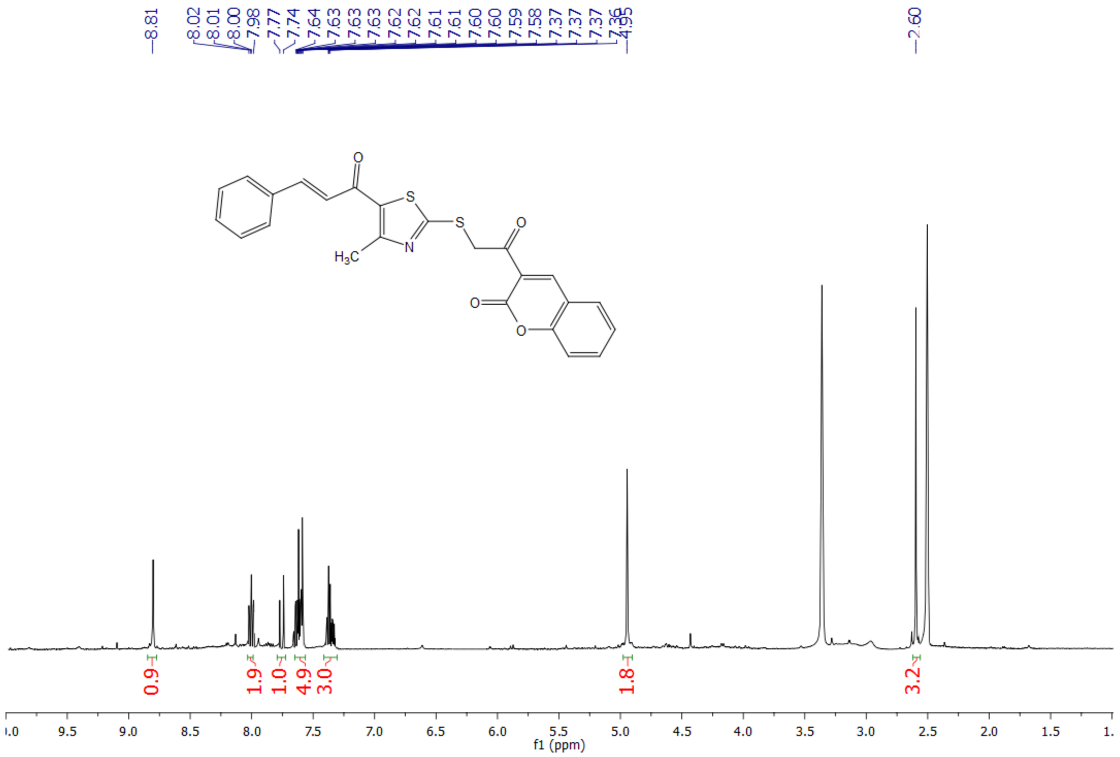

Figure S1. ^{1}H NMR (500 MHz, DMSO-d_6) spectrum of **11a**

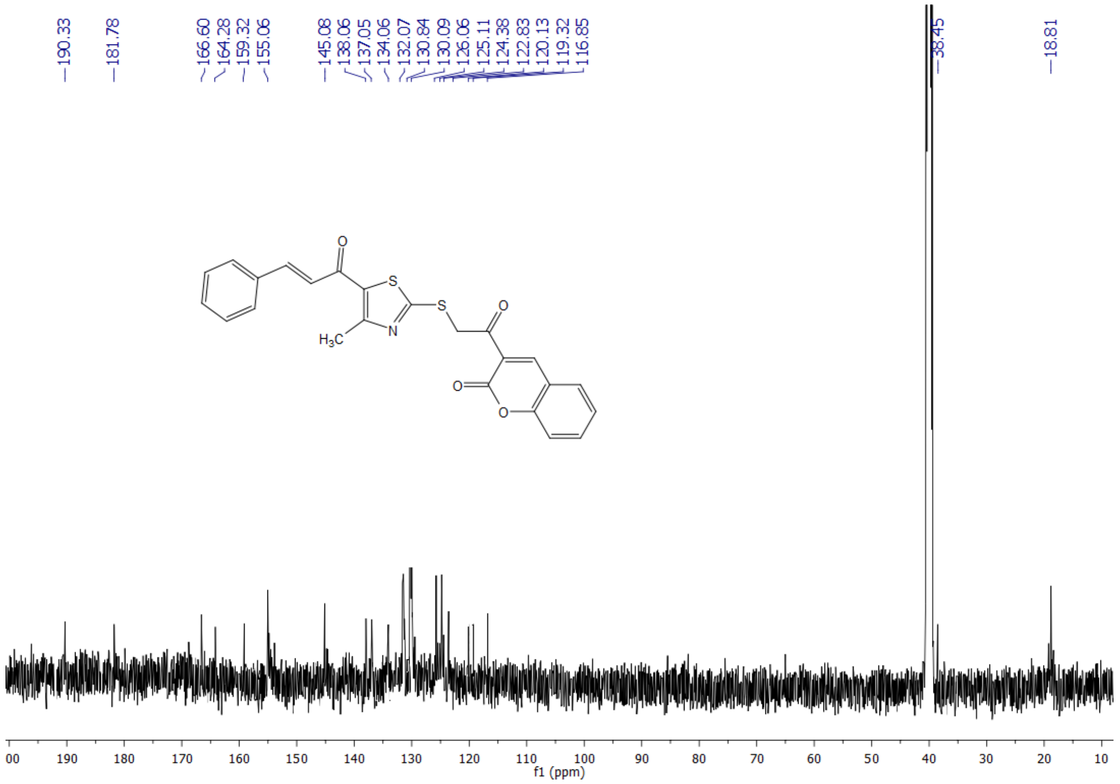

Figure S2. ^{13}C NMR (100 MHz, DMSO-d_6) spectrum of **11a**

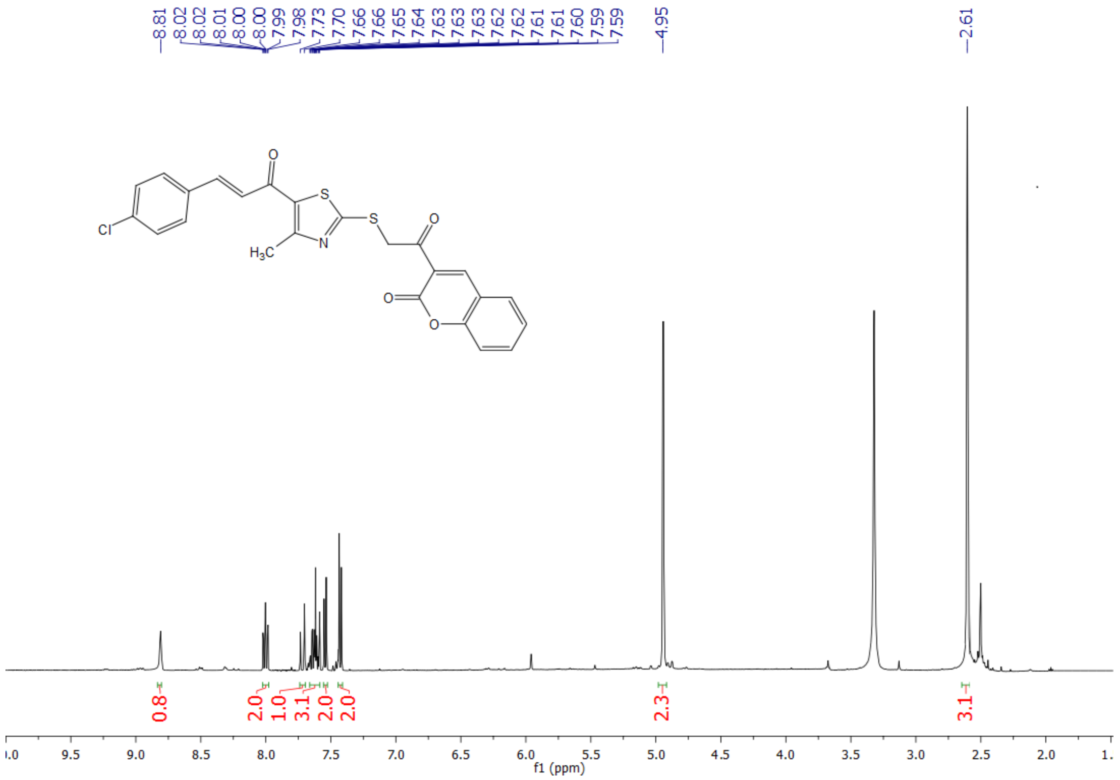

Figure S3. ^{1}H NMR (500 MHz, DMSO-d_6) spectrum of **11b**

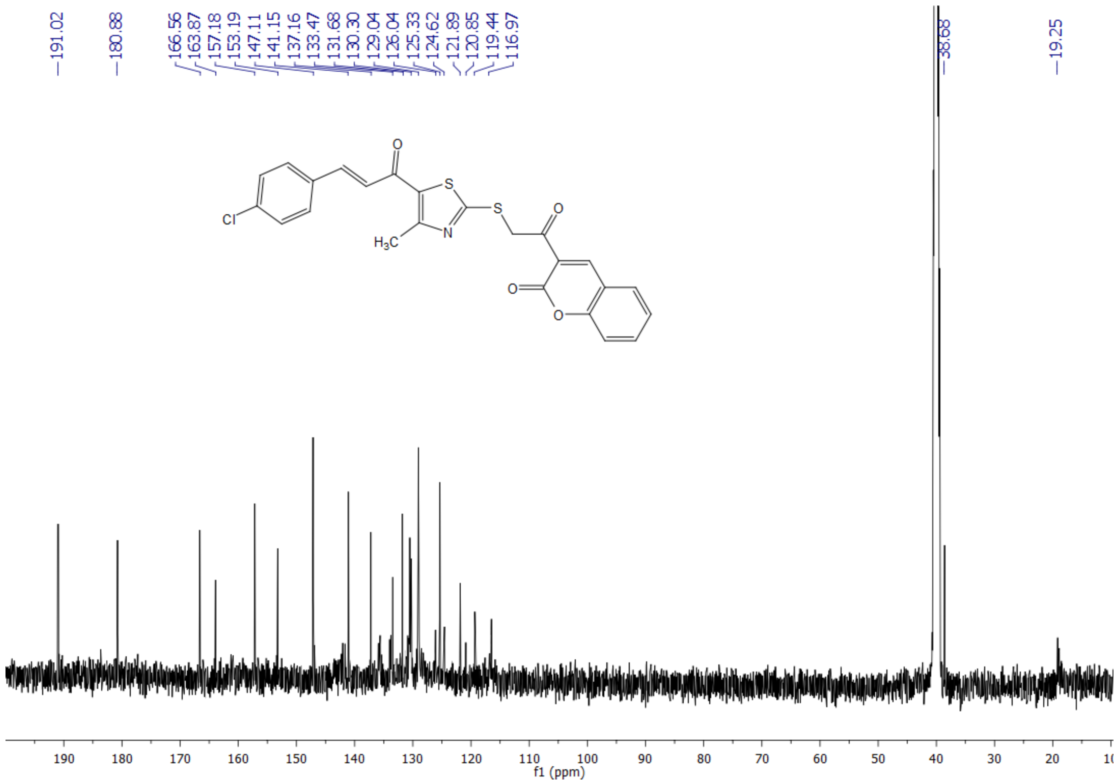

Figure S4. ^{13}C NMR (100 MHz, DMSO-d_6) spectrum of **11b**

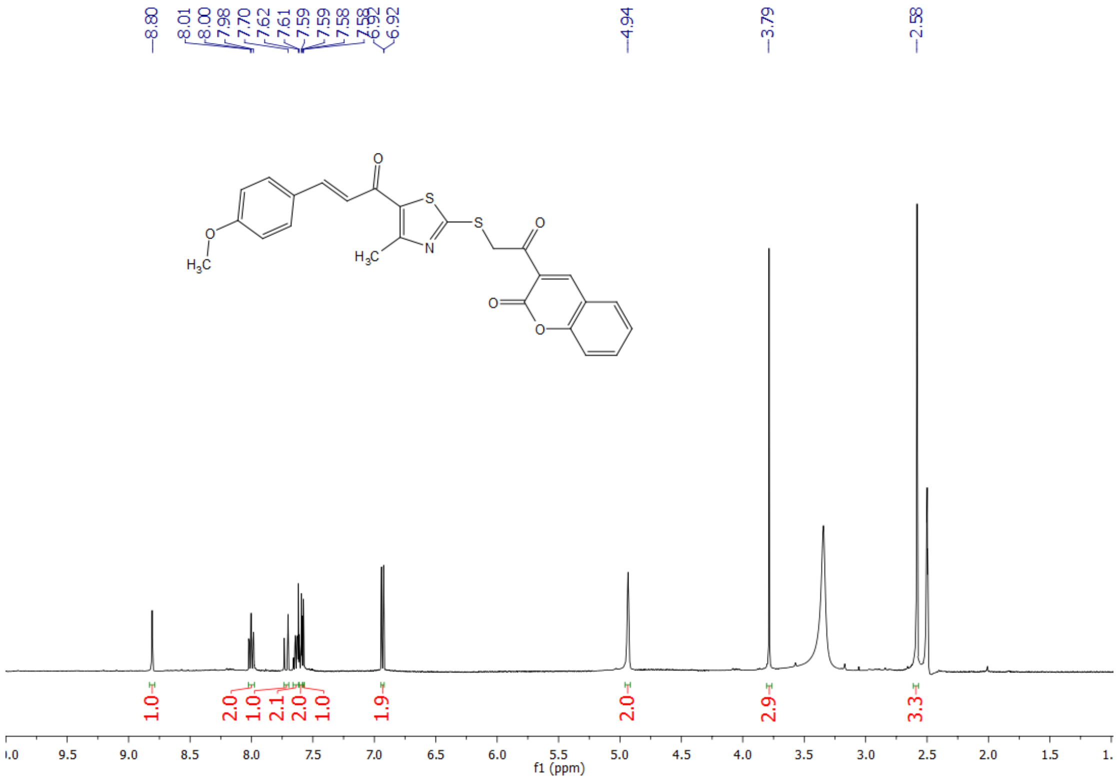

Figure S5. ^{1}H NMR (500 MHz, DMSO-d_6) spectrum of **11c**

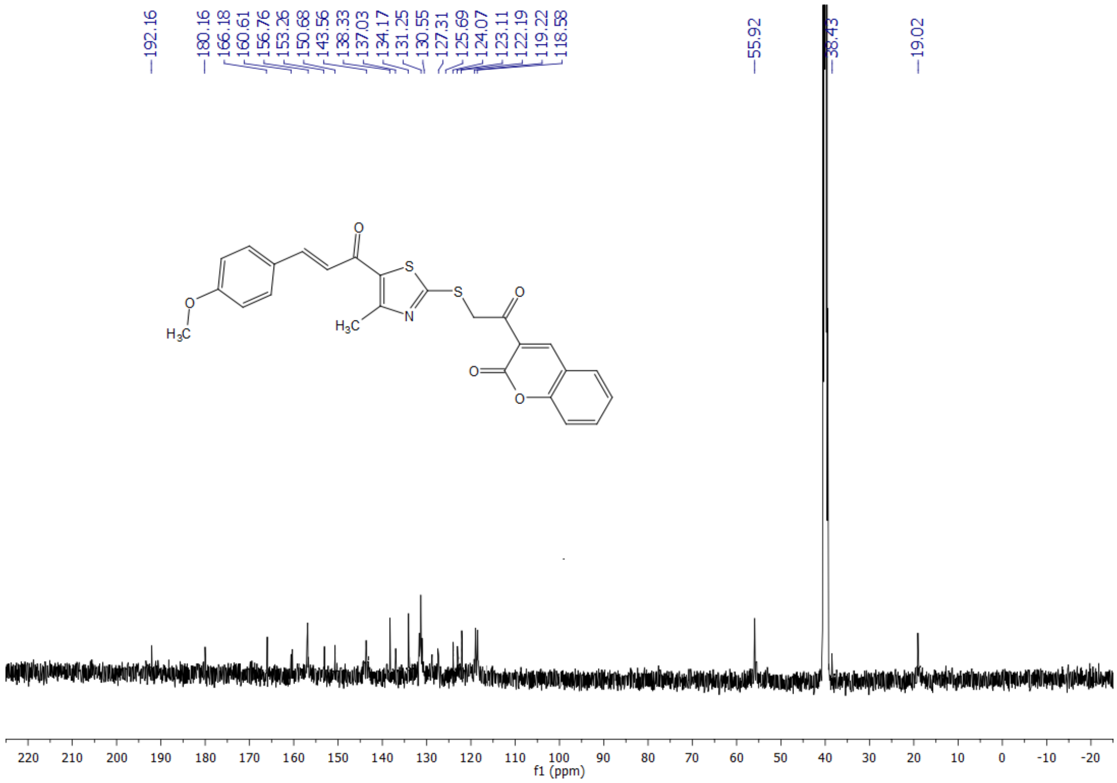

Figure S6. ^{13}C NMR (100 MHz, DMSO-d_6) spectrum of **11c**

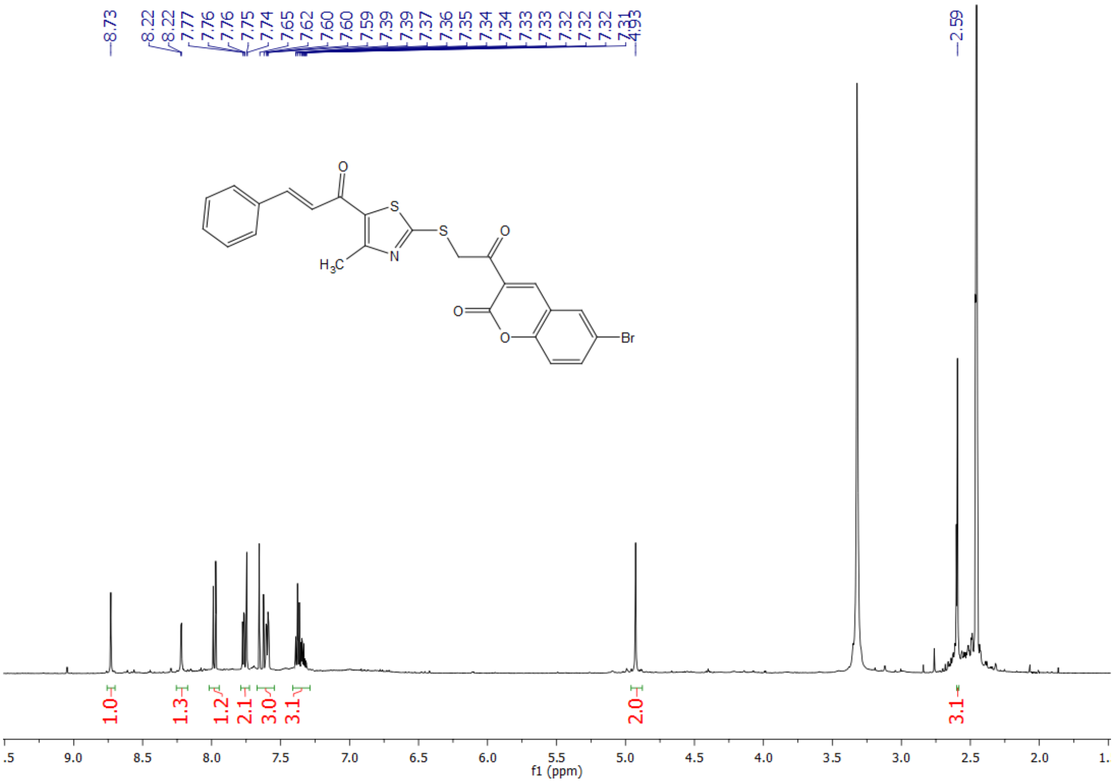

Figure S7. ^{1}H NMR (500 MHz, DMSO-*d*6) spectrum of **11d**

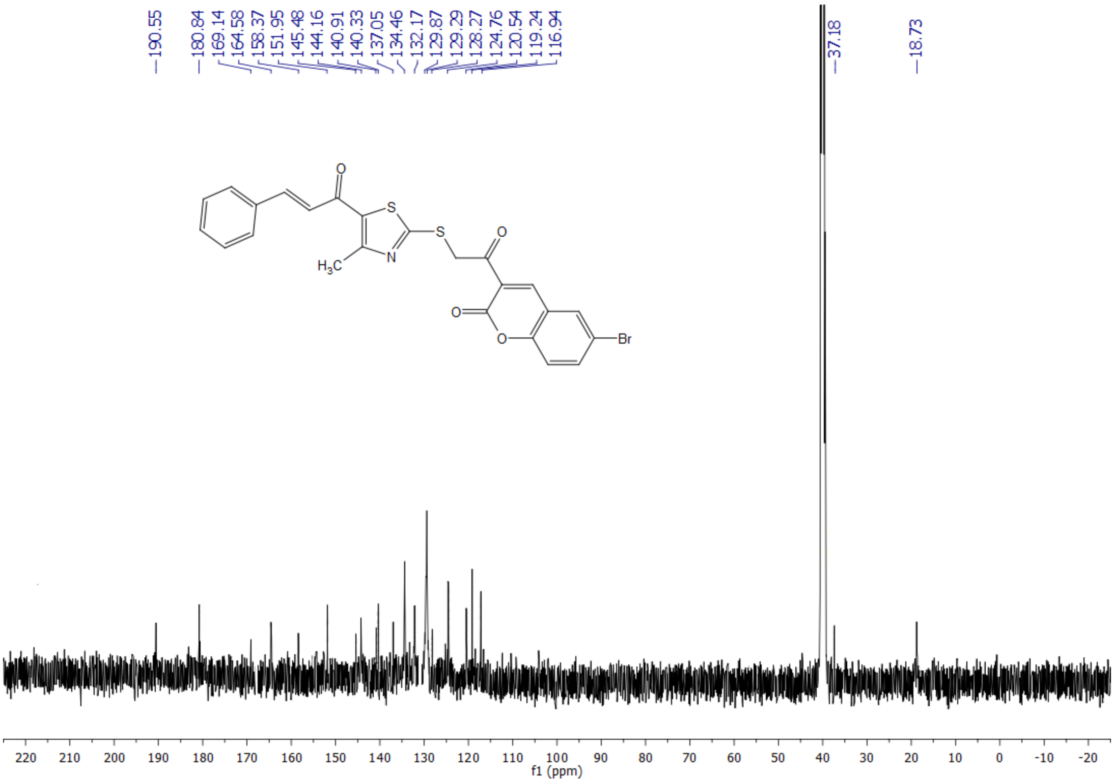

Figure S8. ^{13}C NMR (100 MHz, DMSO-*d*6) spectrum of **11d**

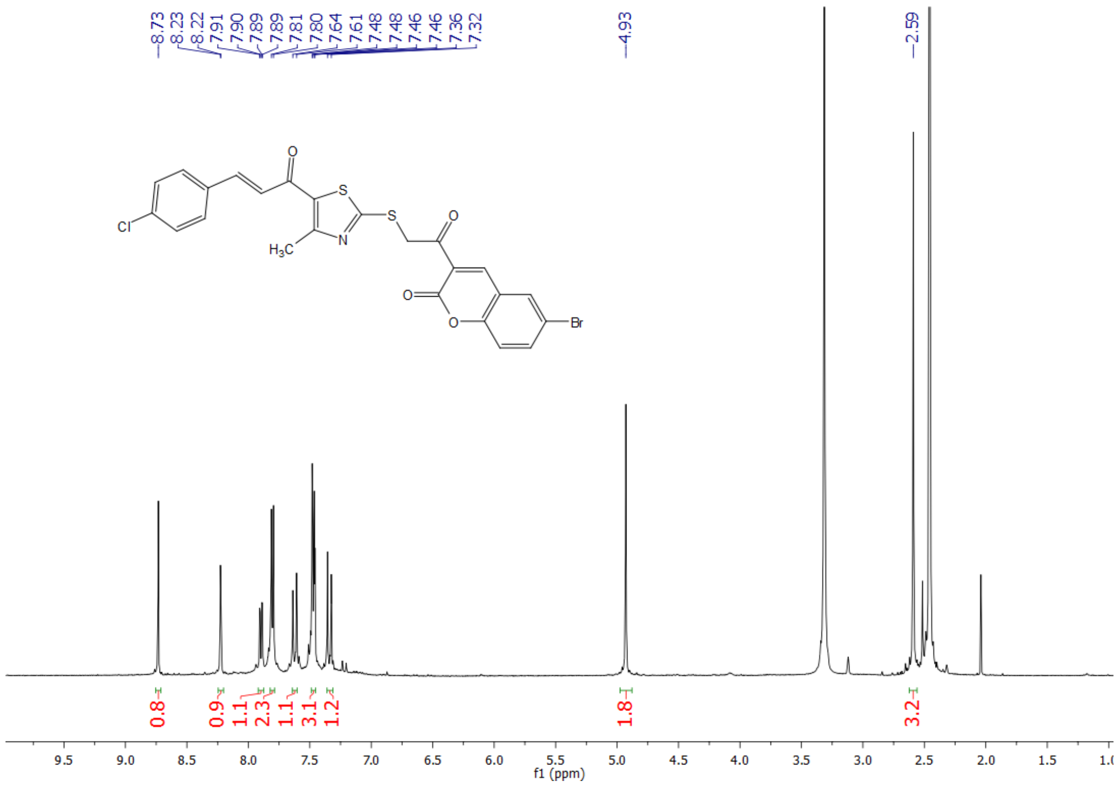

Figure S9. ^{1}H NMR (500 MHz, DMSO-d_6) spectrum of **11e**

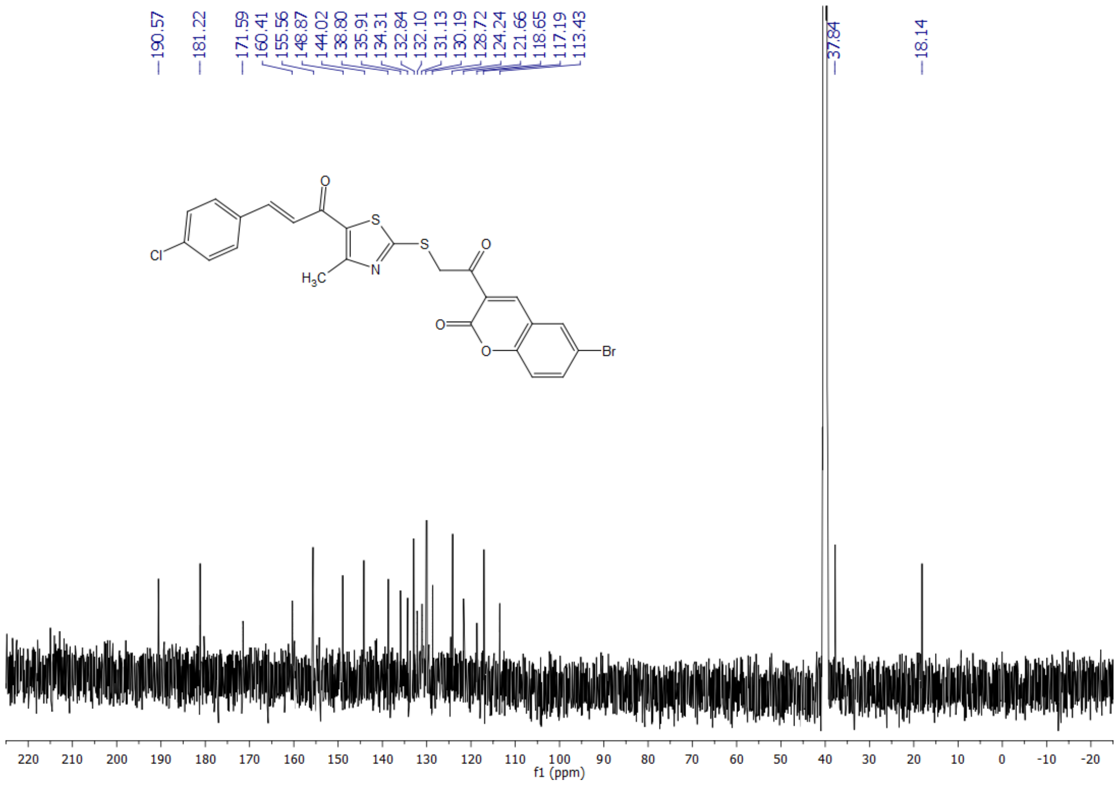

Figure S10. ^{13}C NMR (100 MHz, DMSO-d_6) spectrum of **11e**

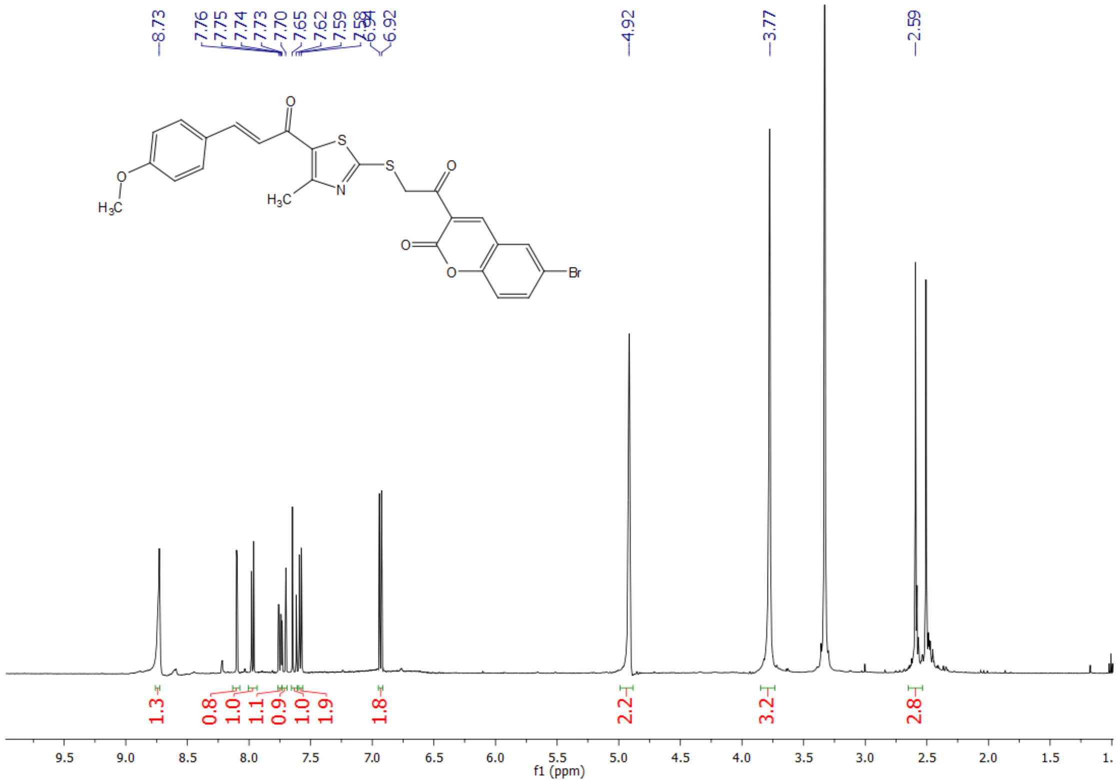

Figure S11. ^{1}H NMR (500 MHz, DMSO-d_6) spectrum of **11f**

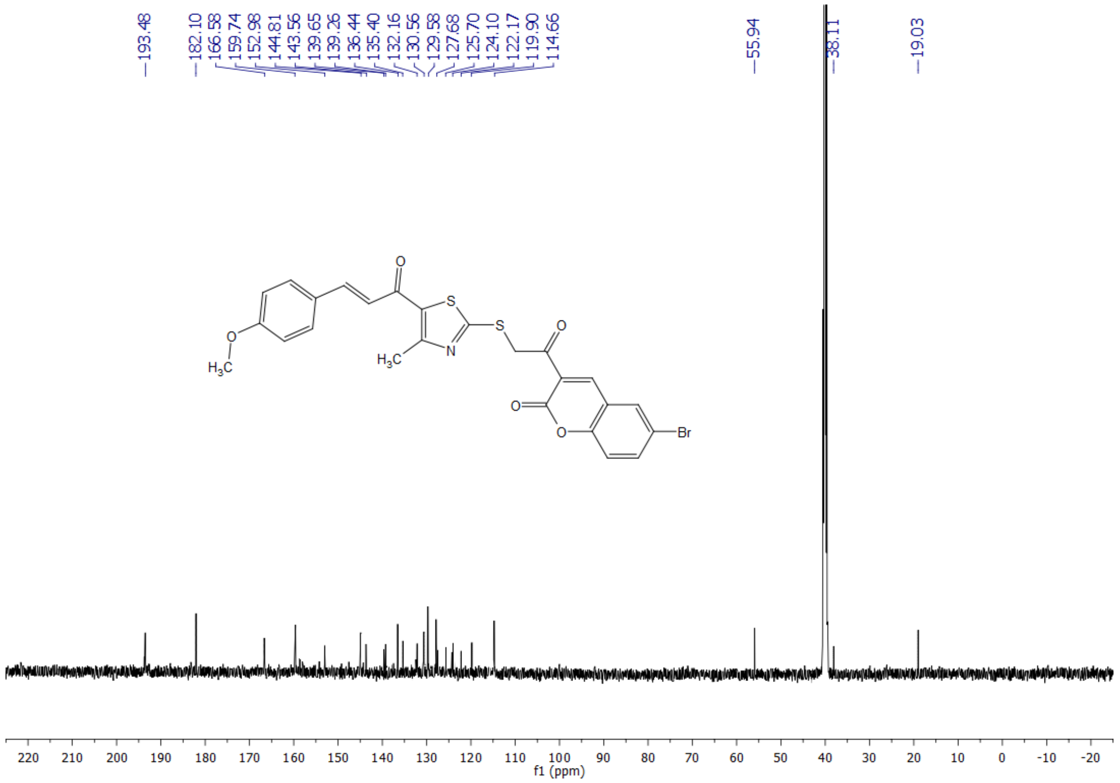

Figure S12. ^{13}C NMR (100 MHz, DMSO-d_6) spectrum of **11f**

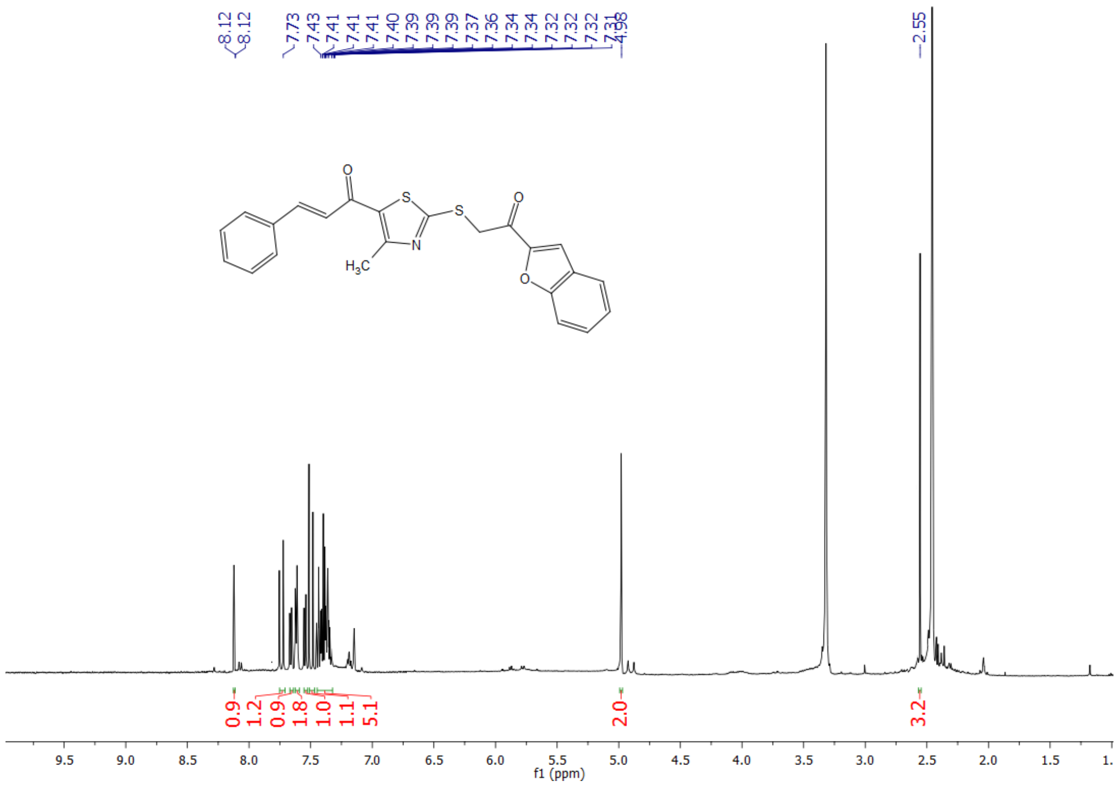

Figure S13. ^{1}H NMR (500 MHz, DMSO-d_6) spectrum of **12a**

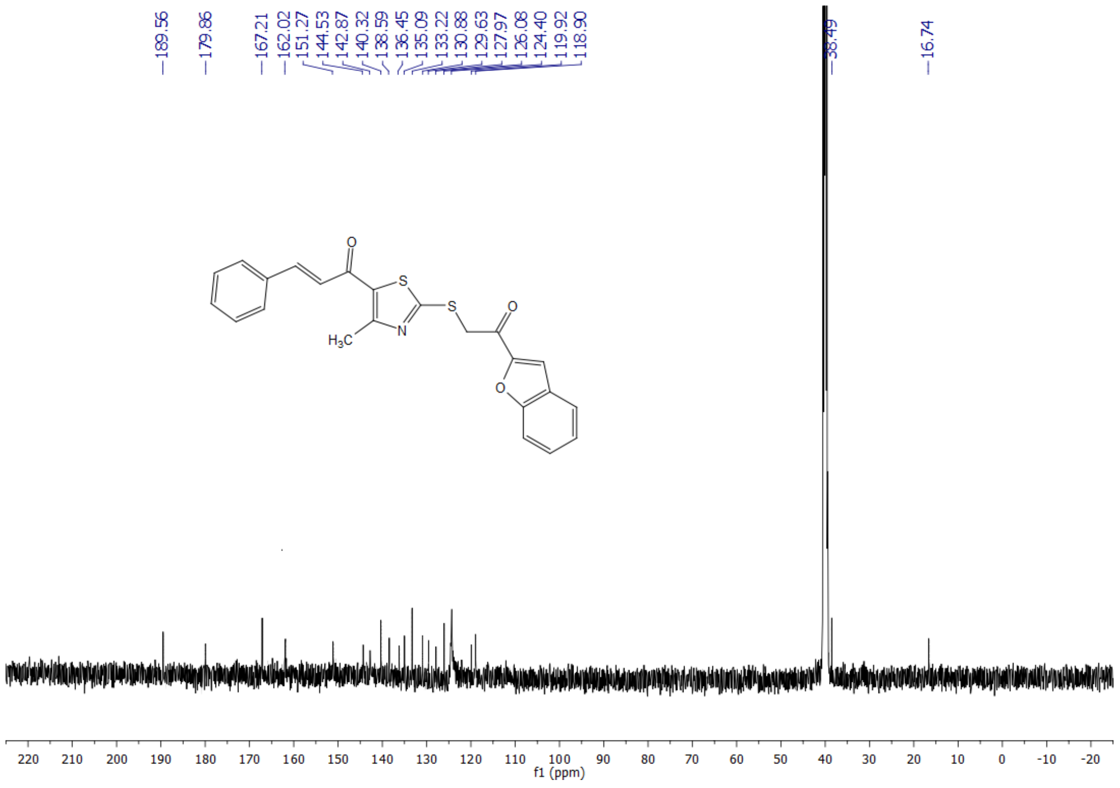

Figure S14. ^{13}C NMR (100 MHz, DMSO-d_6) spectrum of **12a**

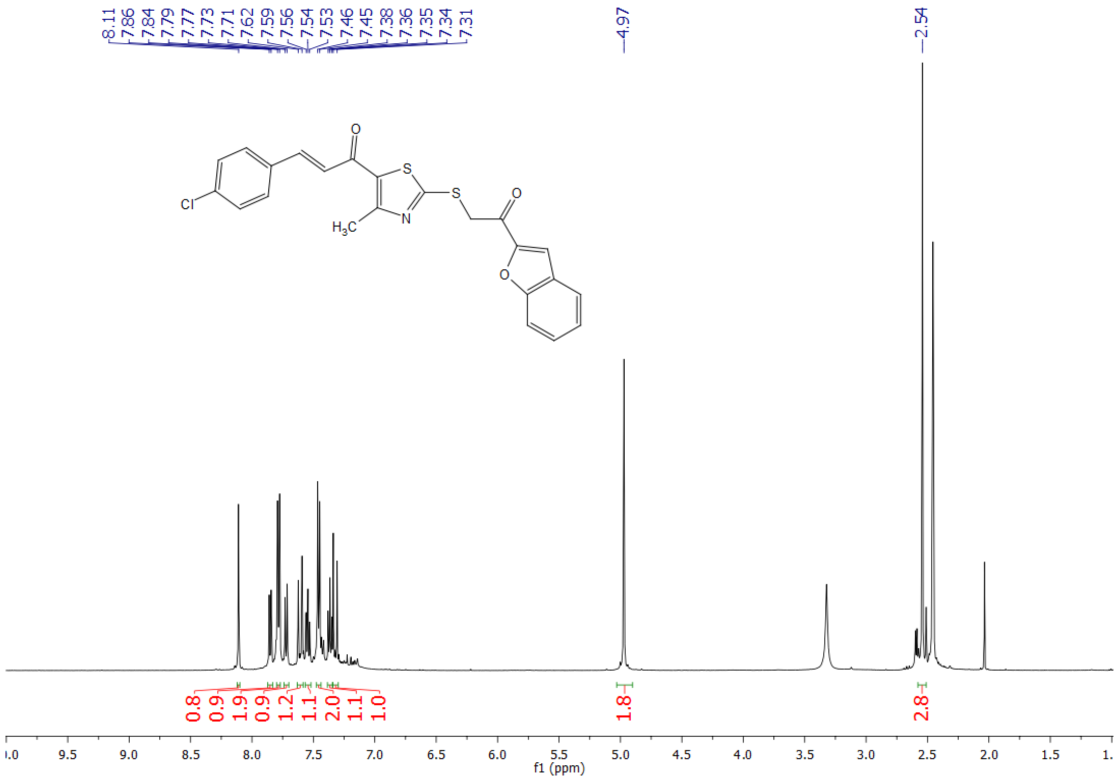

Figure S15. ^{1}H NMR (500 MHz, DMSO-d_6) spectrum of **12b**

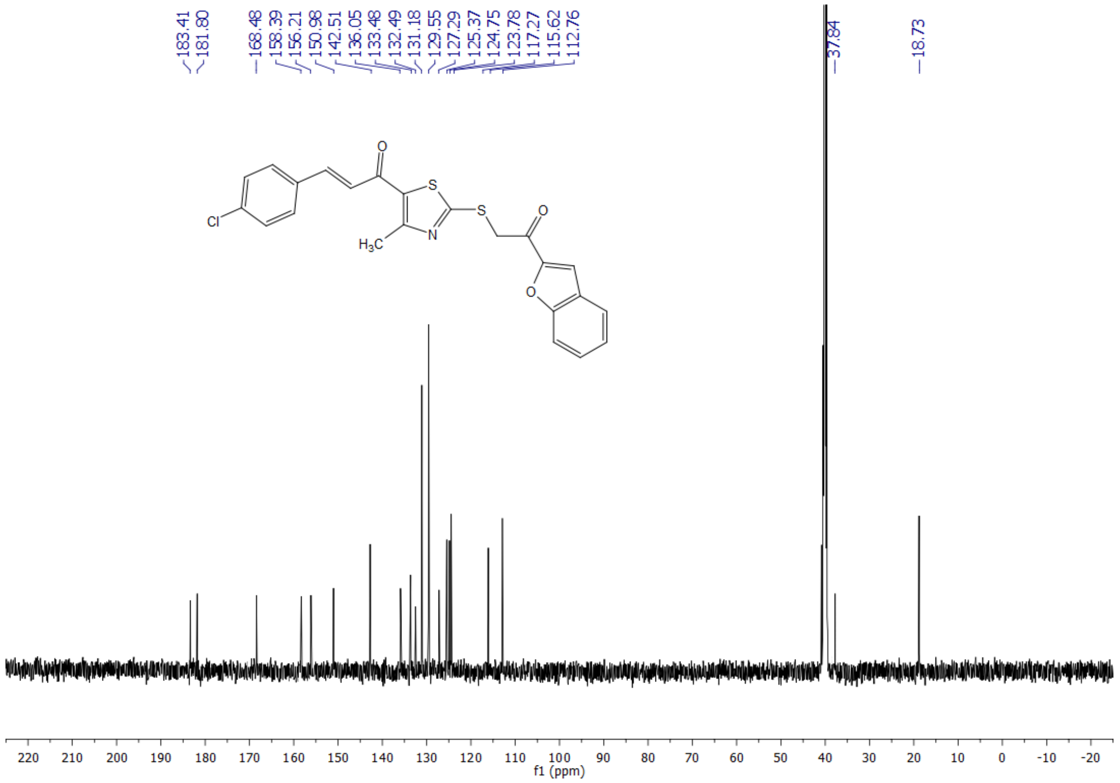

Figure S16. ^{13}C NMR (100 MHz, DMSO-d_6) spectrum of **12b**

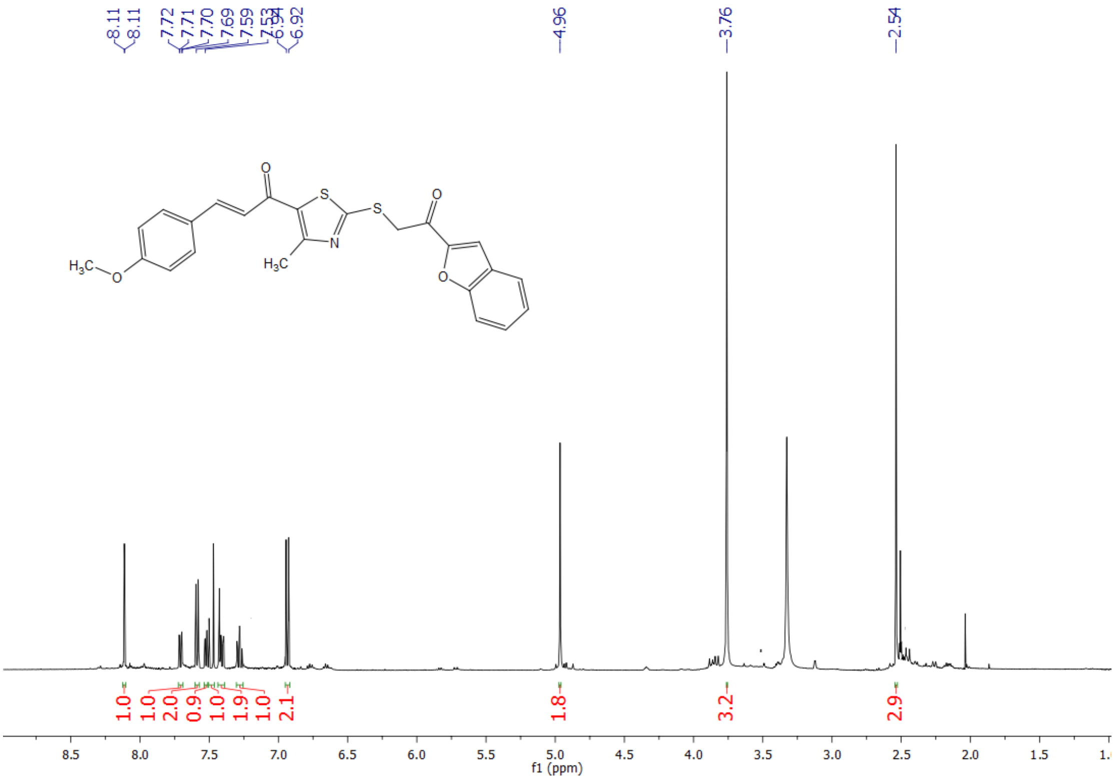

Figure S17. ^{1}H NMR (500 MHz, DMSO-d_6) spectrum of **12c**

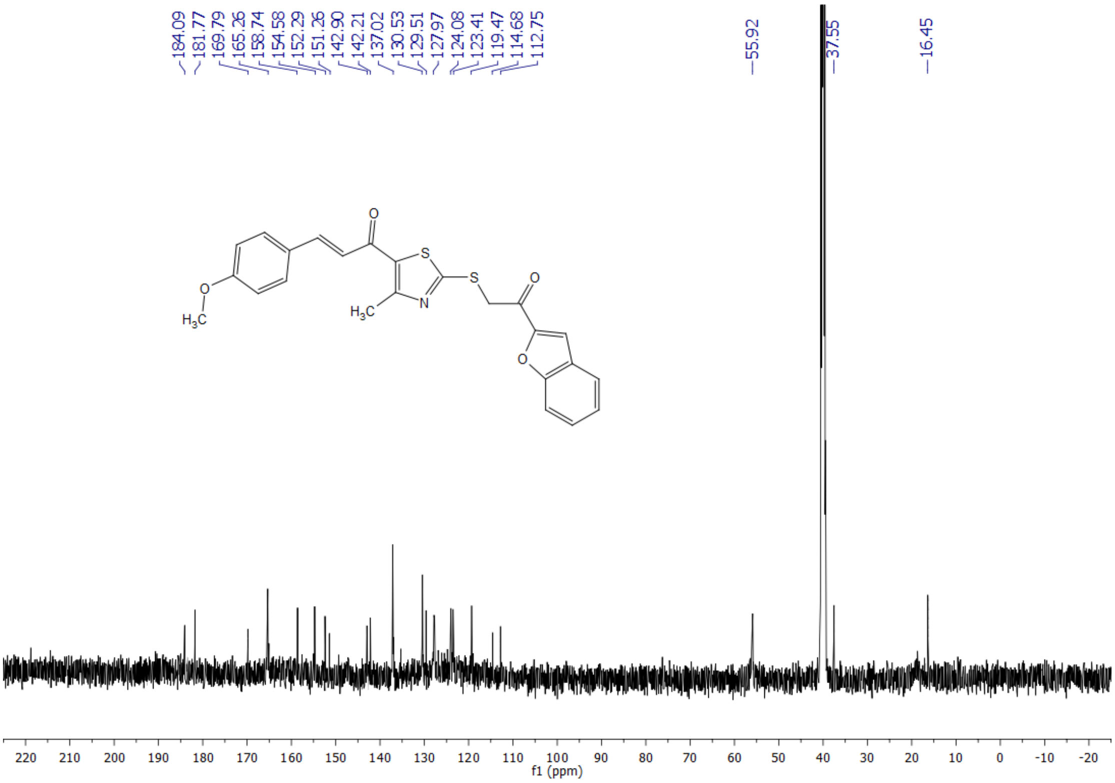

Figure S18. ^{13}C NMR (100 MHz, DMSO-d_6) spectrum of **12c**

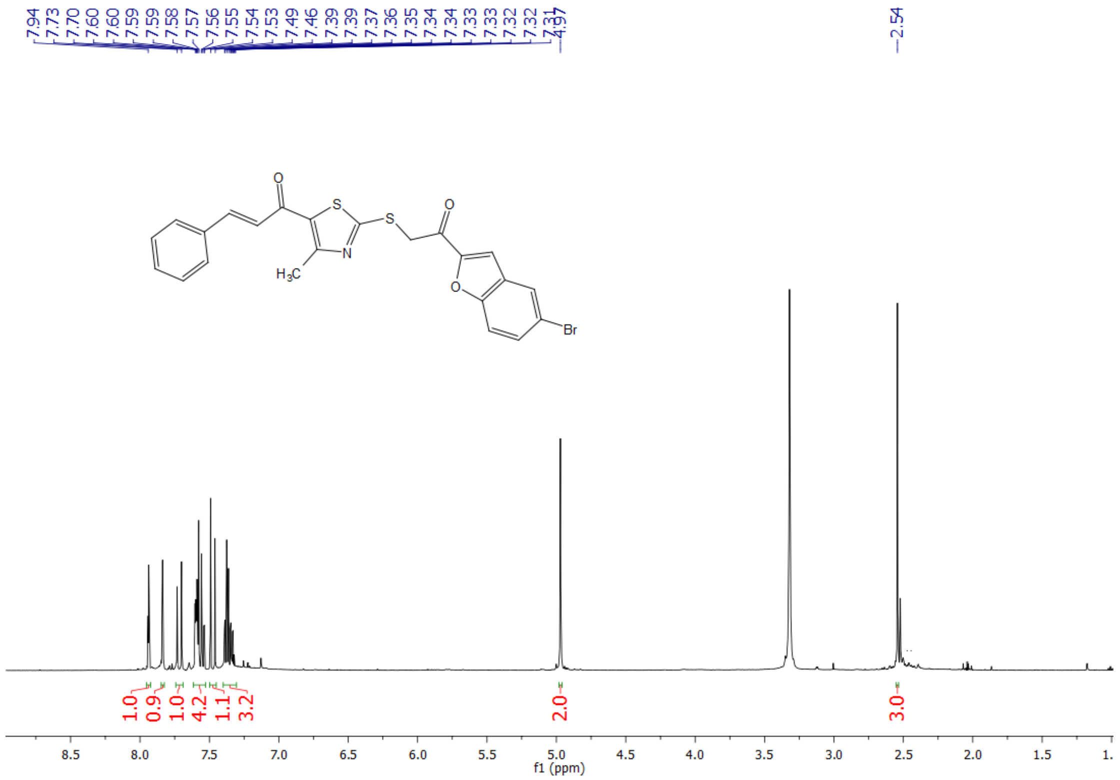

Figure S19. ^{1}H NMR (500 MHz, DMSO-d_6) spectrum of **12d**

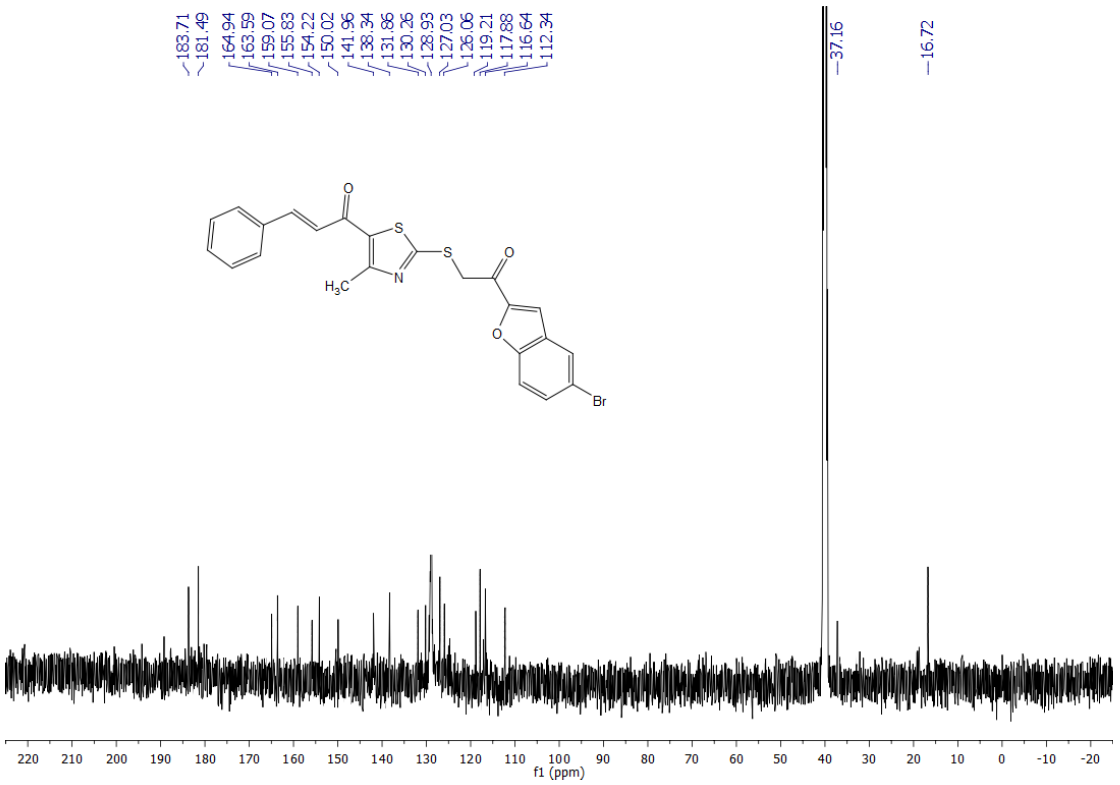

Figure S20. ^{13}C NMR (100 MHz, DMSO-d_6) spectrum of **12d**

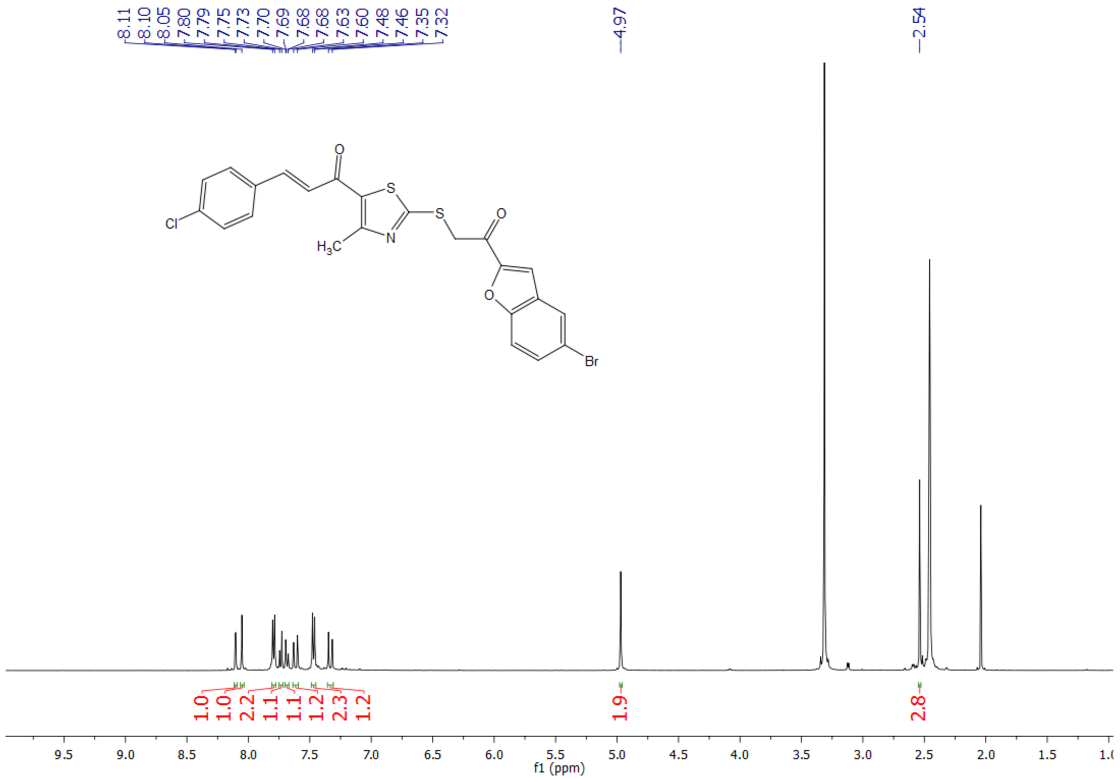

Figure S21. ^{1}H NMR (500 MHz, DMSO-d_6) spectrum of **12e**

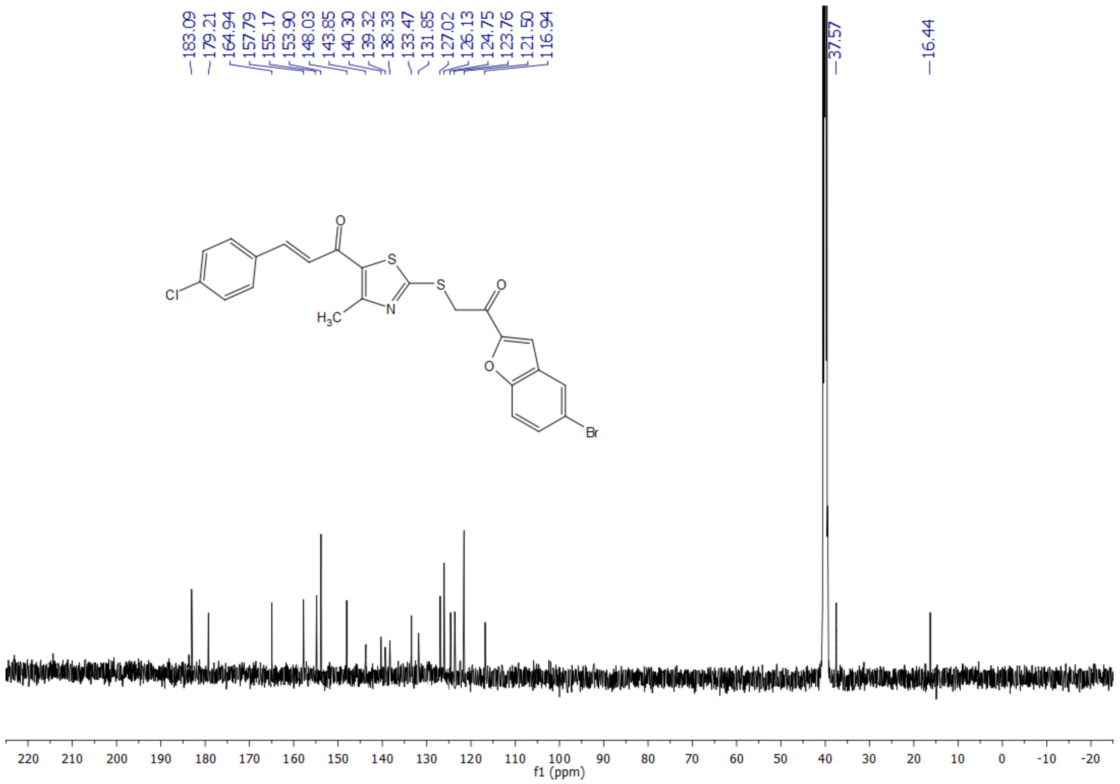

Figure S22. ^{13}C NMR (100 MHz, DMSO-d_6) spectrum of **12e**

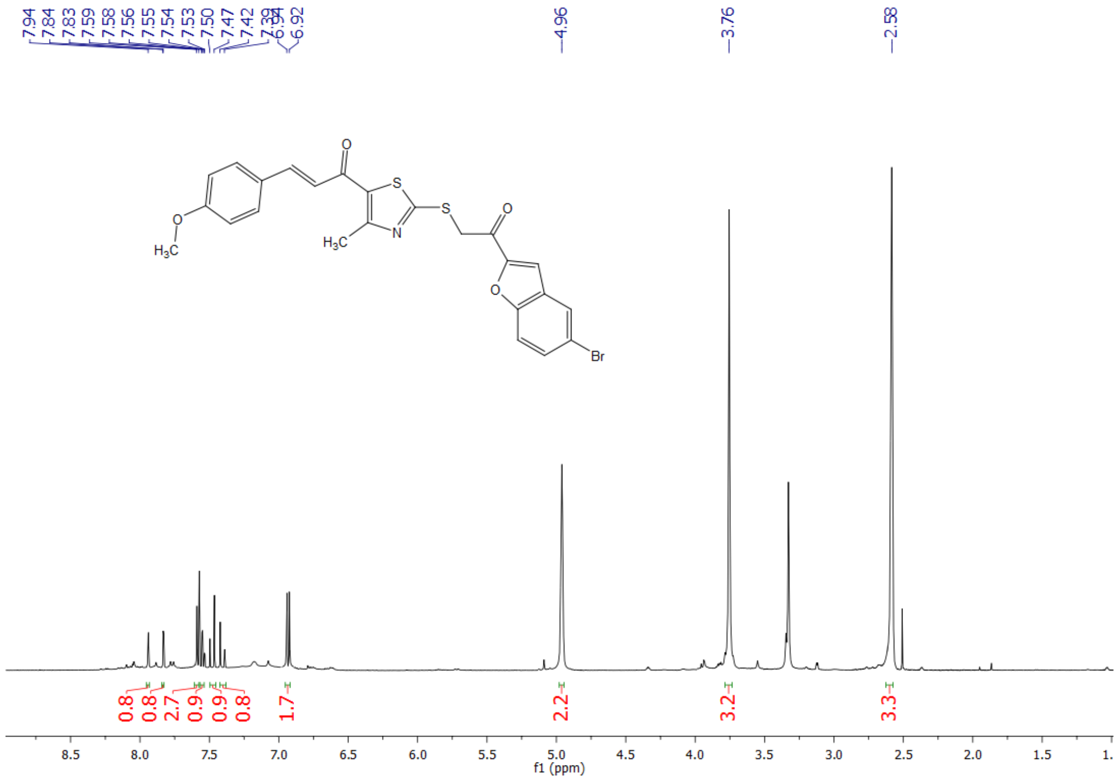

Figure S23. ^{1}H NMR (500 MHz, DMSO-d_6) spectrum of **12f**

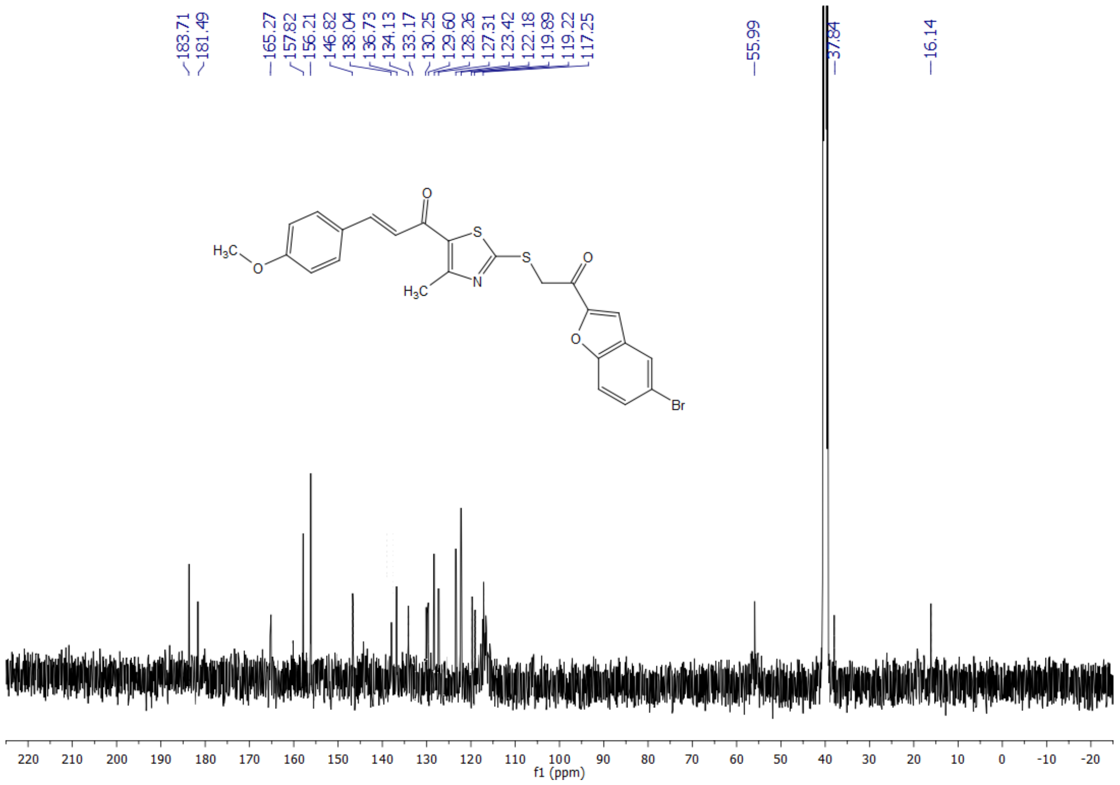

Figure S24. ^{13}C NMR (100 MHz, DMSO-d_6) spectrum of **12f**

Appendix A

4. Experimental

4.1. Chemistry

General Information

All solvents were used as purchased from Merck (St. Louis, MO, USA). The progress of all reactions was monitored with thin-layer chromatography (TLC) on Merck alumina-backed TLC plates and visualized under UV light. Spectra were measured in DMSO-*d6* on a Bruker AV-300 spectrometer (500 MHz for ^{1}H and 100 MHz for ^{13}C), in the Chemistry Department, Faculty of Science, Mansoura University, Egypt. Chemical shifts are expressed in δ (ppm) versus internal Tetramethylsilane (TMS) = 0 ppm for ^{1}H and ^{13}C and the coupling constants are stated in Hz. Splitting patterns are denoted as follows: singlet (s), doublet (d), multiplet (m), triplet (t), quartet (q), doublet of doublets (dd), doublet of triplets (dt), triplet of doublets (td), and doublet of quartet (dq). Melting points (mp) were determined with a Stuart melting point instrument in the Medicinal Chemistry Department, Faculty of Pharmacy, Minia University, El Minia, 61519 Egypt, and are expressed in °C. Elemental analyses were carried out on a Perkin Elmer device at the Microanalytical center, Azhar University, Egypt.

4.2. Biological evaluation

4.2.1 Cell Viability assay (MTT assay)

MTT assay was performed to investigate the effect of the synthesized compounds on mammary epithelial cells (MCF-10A). The cells were propagated in medium consisting of Ham's F-12 medium/ Dulbecco's modified Eagle's medium (DMEM) (1:1) supplemented with 10% foetal calf serum, 2 mM glutamine, insulin (10 μg/mL), hydrocortisone (500 ng/mL) and epidermal growth factor (20 ng/mL). Trypsin ethylenediamine tetra acetic acid (EDTA) was used to passage the cells after every 2-3 days. 96-well flat-bottomed cell culture plates were used to seed the cells at a density of 104 cells mL^{-1}. The medium was aspirated from all the wells of culture plates after 24 h followed by the addition of synthesized compounds (in 200 μL medium to yield a final concentration of 0.1% (v/v) dimethyl sulfoxide) into individual wells of the plates. Four wells were designated to a single compound. The plates were allowed to incubate at 37°C for 96 h. Afterwards, the medium was aspirated and 3-[4,5-dimethylthiazol-2-yl]-2,5-diphenyltetrazolium bromide (MTT) (0.4 mg/mL) in medium was added to each well and subsequently incubated for 3 h. The medium was aspirated and 150 μL dimethyl sulfoxide (DMSO) was added to each well. The plates were vortexed followed by the measurement of absorbance at 540 nm on a microplate reader. The results were presented as inhibition (%) of proliferation in contrast to controls comprising 0.1% DMSO.

4.2.2. Assay for antiproliferative effect

To explore the antiproliferative potential of compounds propidium iodide fluorescence assay was performed using different cell lines such as Panc-1 (pancreas cancer cell line), MCF-7 (breast cancer cell line), HT-29 (colon cancer cell line) and A-549 (epithelial cancer cell line), respectively. To calculate the total nuclear DNA, a fluorescent dye (propidium iodide, PI) is used which can attach to the DNA, thus offering a quick and precise technique. PI cannot pass through the cell membrane and its signal intensity can be considered as directly proportional to quantity of cellular DNA. Cells whose cell membranes are damaged or have changed permeability are counted as dead ones. The assay was performed by seeding the cells of different cell lines at a density of 3000-7500 cells/well (in200µl medium) in culture plates followed by incubation for 24h at 37 °C in humidified 5%CO2/95% air atmospheric conditions. The medium was removed; the compounds were added to the plates at 10 µM concentrations (in 0.1% DMSO) in triplicates, followed by incubation for 48h. DMSO (0.1%) was used as control. After incubation, medium was removed followed by the addition of PI (25 µl, 50µg/mL in water/medium) to each well of the plates. At -80 °C, the plates were allowed to freeze for 24 h, followed by thawing at 25 ^{o}C. A fluorometer (Polar-Star BMG Tech) was used to record the readings at excitation and emission wavelengths of 530 and 620 nm for each well. The percentage cytotoxicity of compounds was calculated using the following formula:

$$\% \text{ Cytotoxicity} = \frac{A_C - A_{TC}}{A_C} \times 100$$

Where A*TC*= Absorbance of treated cells and AC= Absorbance of control. Erlotinib was used as positive control in the assay.

4.2.3. EGFR inhibitory assay

Baculoviral expression vectors including pBlueBacHis2B and pFASTBacHTc were used separately to clone 1.6 kb cDNA coding for EGFR cytoplasmic domain (EGFR-CD, amino acids 645–1186). 5′ upstream to the EGFR sequence comprised a sequence that encoded (His)6. Sf-9 cells were infected for 72h for protein expression. The pellets of Sf-9 cells were solubilized in a buffer containing sodium vanadate (100 μM), aprotinin (10 μg/mL), triton (1%), HEPES buffer(50mM), ammonium molybdate (10 μM), benzamidine HCl (16 μg/mL), NaCl (10 mM),leupeptin (10 μg/mL) and pepstatin (10 μg/mL) at 0°C for 20 min at pH 7.4, followed by centrifugation for 20 min. To eliminate the nonspecifically bound material, a Ni-NTA super flow packed column was used to pass through and wash the crude extract supernatant first with 10mM and then with 100 mM imidazole. Histidine-linked proteins were first eluted with 250 and then with 500 mM imidazole subsequent to dialysis against NaCl (50 mM), HEPES (20 mM), glycerol (10%) and 1 μg/mL each of aprotinin, leupeptin and pepstatin for 120 min. The purification was performed either at 4 °C or on ice. To record autophosphorylation level, EGFR kinase assay was carried out on the basis of DELFIA/Time-Resolved Fluorometry. The compounds were first dissolved in DMSO absolute, subsequent to dilution to appropriate concentration using HEPES (25 mM) at pH 7.4. Each compound (10 μL) was incubated with recombinant enzyme (10 μL, 5 ng for EGFR, 1:80 dilution in 100 mM HEPES) for 10 min at 25°C, subsequent to the addition of 5X buffer (10 μL, containing 2 mM MnCl2, 100 μM Na3VO4, 20 mM HEPES and 1 mM DTT) and ATP-MgCl2 (20 μL, containing 0.1 mM ATP and 50 mM MgCl2) and incubation for 1h. The negative and positive controls were included in each plate by the incubation of enzyme either with or without ATP-MgCl2. The liquid was removed after incubation and the plates were washed thrice using a wash buffer. The Europium-tagged antiphosphotyrosine antibody (75 μL, 400 ng)

was added to each well followed by incubation of 1h and then washing of the plates using buffer. The enhancement solution was added to each well and the signal was recorded at excitation and emission wavelengths of 340 at 615 nm. The autophosphorylation percentage inhibition by compounds was calculated using the following equation:

$$100\% - [(negative\ control)/(positive\ control) - (negative\ control)]$$

Using the curves of percentage inhibition of eight concentrations of each compound, IC50 was calculated. The majority of signals detected by antiphosphotyrosine antibody were from EGFR because the enzyme preparation contained low impurities.

4.2.4. VEGFR-2 inhibitory assay

Prepare all the solutions using autoclaved, deionized water and analytical grade reagents. Prepare and store all the reagents at room temperature (unless indicated otherwise). EDTA solution (0.5 M, pH 8.0): add 95 mL ultra-pure water to 14.612 g EDTA, adjust the pH to 8.0 using NaOH solution, add 5 mL ultra-pure water; 1×Kinase Assay Buffer: add 25 mL HEPES solution (1 M), 190.175 mg EGTA, 5 mL MgCl2 solution (1 M), 1 mL DTT, 50 µL tween-20, 450 mL ultrapure water, adjust pH to 7.5 and constant volume to 500 mL using ultrapure water; 4×Stop solution (40 mM): Mix 0.8 mL of the foregoing EDTA solution, 1 mL 10×Detection Buffer and 8.2 mL ultra-pure water; 1×Detection Buffer: Mix 1 mL 10×Detection Buffer with 9 mL water; 4×VEGFR Kinase solution (1.74 nM, 521 times diluted, stored on ice): 1.2 µL VEGFR mother liquor (0.909 µM) was added to 624 µL 1×Kinase Assay Buffer and mixed; 4×ULight™-labeled JAK1 (substrate) (200 nM, 25 times diluted): 24 µL ULight™-labeled JAK1 (mother liquor concentration 5 µM) was added to 576 µL 1×Kinase Assay Buffer and mixed; 4×ATP Solution (40 µM, 250 times diluted): add 3 µL ATP solution (10 mM) to 747 µL 1×Kinase Assay Buffer

and mixed; 4×Detection Mix (8 nM, 390.6 times diluted): 3 μL Europium- antiphospho-tyrosine antibody (PT66) (3.125 μM) was added to 1169 μL1×Detection Buffer and mixed; 2×substrate/ATP Mix: 560 μL foregoing 4×ULight™-labeled JAK1 and 560 μL 4×ATP solution and mixed (prepared before use). The assays used an ULight-labeled peptide substrate and a Europium-W1024-labeled antiphosphotyrosine antibody. The VEGFR-2 was purchased from Carna Biosciences, Inc. (New York, USA). The 384-well plates were obtained from PerkinElmer. Compounds were dissolved in DMSO and diluted to 11 concentrations at a tripling rate from 2.500 μM to 0.042 nM and added 2.5 μL to 384-well plates. 5 μL 2×VEGFR-2 kinase solution (0.5 nM) was added to 384-well plates homogeneous mixing and pre-reaction at room temperature

for 30 min. Next, 2.5 μL 4×Ultra ULightTM-JAK-1(Tyr1023) Peptide (200 nM)/ATP (40 μM) was added to the corresponding wells of a 384-well plate. Negative control: 2.5 μL/well 4×substrate/ATP mixture and 7.5 μL 1×kinase assay buffer in 384-well plate well. Positive control: 2.5 μL/well 4×substrate/ATP mixture, 2.5 μL/well 1×kinase assay buffer with 16% DMSO, 5 μL/well 2×VEGFR-2 kinase solution was added to the 384-well plate, The final concentration of DMSO in the mixing system was 4%. After incubation at room temperature and dark for 60 min, 5 μL 4×stop solution was added to corresponding wells to react for 5 min and then 5 μL 4×detection mix was added to the corresponding wells of a 384-well plate. The mixture was centrifugally mixed and stayed for 60 min at room temperature for color development. The plate was read using a Envision plate reader. The inhibition rate (%) = (positive well reading-compound well reading)/(positive well reading-negative well reading)×100. The corresponding IC_{50} values were calculated using GraphPad Prism 5.0.

4.2. Antimicrobial activity

4.2.1. Organisms and culture conditions

The cultures used were collected from the Cairo University's Microanalytical Centre, Faculty of Science. An updated Kirby-Bauer disc diffusion method was applied for antimicrobial activities of the tested compounds. Shortly, the 10 ml of fresh medium was grown to 100 µl bacteria / food until a count of 108 cell / ml or 105 cell / ml was achieved. 100 µl microbial suspension has been spread over agar plates that suit the broth in which it was held. Selected colonies of each organism that may play a pathogenic function should be from the primary agar plates and tested by the disc diffusion method for susceptibility. Plates inoculated with filamentous fungi *as Aspergillus flavus* at 25°C for 48 hours; Gram positive bacteria as *Staphylococcus aureus* (ATCC 12600), *Bacillus subtilis* (ATCC 6051); Gram negative bacteria as *Escherichia coli* (ATCC 11775), *Pseudomonas aeuroginosa* (ATCC 10145) they were incubated at 35-37°C for 24-48 hours and, then the diameters of the inhibition zones were measured in millimeters. Standard discs of ciprofloxacin (Antibacterial agent), but filter discs impregnated with 10 µl of solvent (distilled water, chloroform, DMSO) were used as a negative control. Blank paper disks (Schleicher & Schuell, Spain) with a diameter of 8.0 mm were impregnated 10µ of tested concentration of the stock solutions. When a filter paper disc impregnated with a tested chemical is placed on agar the chemical will diffuse from the disc into the agar. This diffusion will place the chemical in the agar only around the disc. The solubility of the chemical and its molecular size will determine the size of the area of chemical infiltration around the disc. If an organism is placed on the agar, it will not grow in the area around the disc if it is susceptible to the chemical. This area of no growth around the disc is known as a “Zone of inhibition” or" Clear zone". For the disc diffusion, the zone diameters were measured with slipping calipers of the National Committee for Clinical Laboratory

Standards, and the results are given in **Table 3**. Agar-based methods such as E-test and disk diffusion can be good alternatives because they are simpler and faster than broth-based methods.

4.2.2. Minimum inhibitory concentration assay

In 96-well microtiter plates and 50 mL of fresh bacterial culture of a single McFarland unit overnight, a double serial dilution of each compound (100 mL) in sterile standard saline were prepared to every single source well. Ciprofloxacin antibiotic (5 mg / mL-1) and normal saline were included as standard reference in each assay. The plates were incubated at 37 ^{0}C overnight. As an indicator of bacterial growth, 40 mL of p-iodonitrotetrazolium violet (INT) was added to each well and incubated at 37 ^{0}C for 30 min. MIC values are recorded as the lowest concentration of the extract that completely inhibited bacterial growth that is clear well. The colorless tetrazolidium salt acts as an electron accepter and is reduced to a red colored formazan product by biological activity organisms. Where bacterial growth was inhibited, the solution in the well remained clear after incubation with INT. The observed MIC values are presented in **Table 1**.

4.2.3. Determination of Inhibitory Activities on *E. coli* DNA Gyrase.

All the final compounds were tested for *E. coli* DNA gyrase inhibitory activity in a supercoiling assay. Activities were determined on streptavidin-coated 96-well microtiter plates from Thermo scientific Pierce. First, the plates were rehydrated with buffer (20 mM Tris-HCl with pH 7.6, 0.01% w/v BSA, 0.05% v/v Tween 20, 137 mM NaCl) and the biotinylated oligonucleotide was then immobilized. After washing off the unbound oligonucleotide, the enzyme test was performed. The reaction volume of 30 µL in buffer (35 mM Tris-HCl with pH 7.5, 4 mM $MgCl_2$, 24 mM KCl, 2 mM DTT, 1.8 mM spermidine, 1 mM ATP, 6.5 % w/v glycerol, 0.1 mg/mL albumin) contained 1.5 U of DNA gyrase from *E. coli* or *S. aureus*, 0.75 µg of relaxed pNO1 plasmid, and 3 µL solution of the inhibitor in 10% DMSO and 0.008% Tween 20. Reaction solutions were incubated at 37 °C

for 30 min. After that, the TF buffer (50 mM NaOAc with pH 5.0, 50 mM NaCl and 50 mM $MgCl_2$) was added to terminate the enzymatic reaction. After additional incubation for 30 min at rt, during which biotin-oligonucleotide-plasmid triplex was formed, the unbound plasmid was washed off using TF buffer and SybrGOLD in T10 buffer (10 mM Tris HCl with pH 8.0 and 1 mM EDTA) was added. The fluorescence was measured with a microplate reader (BioTek Synergy H4, excitation: 485 nm, emission: 535 nm). Initial screening was done at 100 or 10 μM concentration of inhibitors. For the most active inhibitors IC_{50} was determined using seven concentrations of tested compounds. GraphPad Prism software was used to calculate the IC_{50} values. The result is given as the average value of three independent measurements. As the internal standard novobiocin (IC_{50} = 0.168 μM for *E. coli* gyrase and IC_{50} = 0.041 μM for *S. aureus* gyrase) was used. Determination of inhibitory activities on *E. coli* and *S. aureus* Topoisomerase IV. IC_{50} values were determined in an assay from In spiralis on streptavidin-coated 96-well microtiter plates from Thermo scientific Pierce. First, the plates were rehydrated with buffer (20 μM Tris-HCl with pH 7.6, 0.01% w/v BSA, 0.05% v/v Tween 20, 137 mM NaCl) and biotinylated oligonucleotide was then immobilized. After washing off the unbound oligonucleotide, the enzyme test was performed. The reaction volume of 30 μL in buffer (40 mM HEPES KOH with pH 7.6, 100 mM potassium glutamate, 10 mM magnesium acetate, 10 mM DTT, 1 mM ATP, 0.05 mg/mL albumin) contained 1.5 U of topoisomerase IV from *E. coli* or *S. aureus*, 0.75 μg of pNO1 supercoiled plasmid, and 3 μL solution of the inhibitor in DMSO (10%) and Tween 20 (0.008%). Reaction mixtures were incubated at 37 °C for 30 min and after that, the TF buffer (50 mM NaOAc with pH 5.0, 50 mM NaCl and 50 mM $MgCl_2$) was added to terminate the enzymatic reaction. After additional incubation for 30 min at rt, during which triplex (biotin-oligonucleotide-plasmid) was formed, the unbound plasmid was washed off using TF buffer and Sybr GOLD in T10 buffer (10 mM Tris HCl

with pH 8.0 and 1 mM EDTA) was added. The fluorescence was measured with a microplate reader (BioTek Synergy H4, excitation: 485 nm, emission: 535 nm). Initial screening was done at 100 or 10 μM concentration of inhibitors. For the most active inhibitors IC_{50} was determined using seven concentrations of tested compounds. GraphPad Prism software was used to calculate the IC_{50} values. The result is given as the average value of three independent measurements. As the internal standard novobiocin (IC_{50} = 11.1 μM) for *E. coli* topoisomerase IV and IC_{50} = 26.7 μM for *S. aureus* topoisomerase IV) was used.

4.3. Molecular modeling

Molecular Docking:

The crystal structures of EGFR kinase domain (PDB code: 5D41), VEGFR2 kinase (PDB code: 3U6J) domain and *E. coli* DNA Gyrase (PDB code: 3G7E) were downloaded from the Protein Data Bank. Structures of compounds **11f** and **11b** were drawn and optimized using Marvin-Sketch and Avogadro molecular editors. The proteins were prepared using autodock tools where the co-crystallized ligands and water molecules were removed then kollman charges and polar hydrogens were added. The grid dimensions were set to 80x80x80. Autodock vina was used for molecular docking and the best docking poses were visualized using Discovery Studio Visualizer.

4.4. Statistical analysis

Computerized Prism 5 program was used to statistically analyzed data using one-way ANOVA test followed by Tukey's as post ANOVA for multiple comparison at P ≤.05. Data were presented as mean ± SEM.
